# The Effects of the Exposure of Musculoskeletal Tissue to Extracorporeal Shock Waves

**DOI:** 10.3390/biomedicines10051084

**Published:** 2022-05-06

**Authors:** Tobias Wuerfel, Christoph Schmitz, Leon L. J. Jokinen

**Affiliations:** Extracorporeal Shock Wave Research Unit, Chair of Neuroanatomy, Institute of Anatomy, Faculty of Medicine, Ludwig-Maximilians-University of Munich, Munich 80336, Germany; t.wuerfel@campus.lmu.de (T.W.); leon.jokinen@campus.lmu.de (L.L.J.J.)

**Keywords:** extracorporeal shock wave therapy, ESWT, focused extracorporeal shock wave therapy, fESWT, mechanisms of action, radial extracorporeal shock wave therapy, rESWT, systematic review

## Abstract

Extracorporeal shock wave therapy (ESWT) is a safe and effective treatment option for various pathologies of the musculoskeletal system. Many studies address the molecular and cellular mechanisms of action of ESWT. However, to date, no uniform concept could be established on this matter. In the present study, we perform a systematic review of the effects of exposure of musculoskeletal tissue to extracorporeal shock waves (ESWs) reported in the literature. The key results are as follows: (i) compared to the effects of many other forms of therapy, the clinical benefit of ESWT does not appear to be based on a single mechanism; (ii) different tissues respond to the same mechanical stimulus in different ways; (iii) just because a mechanism of action of ESWT is described in a study does not automatically mean that this mechanism is relevant to the observed clinical effect; (iv) focused ESWs and radial ESWs seem to act in a similar way; and (v) even the most sophisticated research into the effects of exposure of musculoskeletal tissue to ESWs cannot substitute clinical research in order to determine the optimum intensity, treatment frequency and localization of ESWT.

## 1. Introduction

Extracorporeal shock wave therapy (ESWT) is a safe and effective treatment option for various pathologies of the musculoskeletal system. The beginning of the use of extracorporeal shock waves (ESWs) in medicine was in kidney stone fragmentation; the corresponding method is called Extracorporeal Shock Wave Lithotrypsy (ESWL). After ESWL was performed on dogs for the first time in 1976, four years later, the first human patient was successfully freed from his kidney stone disease using ESWL [1]. Expanded to other stone diseases in the gallbladder [2], pancreas [3], bile duct [4] and salivary glands [5], urologists found (more or less by chance) that the application of ESWs in the area of ureteral stones caused changes in the os ileum [6]. Specifically, when bones were exposed to ESWs, primary osteocyte damage followed by osteoblast stimulation was observed [6]. This resulted in the demonstration of the stimulation of fracture healing with ESWs in animal models [7]. Since these beginnings, the application of ESWs has been expanded to a variety of pathologies of the musculoskeletal system, with the treatment of non-unions (reviewed in [8]) and tendinopathies (reviewed in [9,10,11]) being, by far, the largest groups of indications. The treatment of pathologies of the musculoskeletal system with ESWs is commonly referred to as Extracorporeal Shock Wave Therapy (ESWT) and is thus distinguished from ESWL.

This short description of the history of ESWT demonstrates that the development of this treatment modality has not followed the classical drug discovery process, from initial target identification and validation, through assay development, high throughput screening, hit identification, lead optimization and finally the selection of a candidate molecule for clinical development [12]. Rather, progress in clinical research on ESWT was either accompanied or followed by basic and preclinical research into the potential mechanisms of action of ESWs on the target tissue. The latter was addressed in several recent reviews (e.g., [13,14,15,16,17]). Considering the fact that this study summarizes and discusses 181 studies addressing the effects of the exposure of musculoskeletal tissue on ESWs [6,18,19,20,21,22,23,24,25,26,27,28,29,30,31,32,33,34,35,36,37,38,39,40,41,42,43,44,45,46,47,48,49,50,51,52,53,54,55,56,57,58,59,60,61,62,63,64,65,66,67,68,69,70,71,72,73,74,75,76,77,78,79,80,81,82,83,84,85,86,87,88,89,90,91,92,93,94,95,96,97,98,99,100,101,102,103,104,105,106,107,108,109,110,111,112,113,114,115,116,117,118,119,120,121,122,123,124,125,126,127,128,129,130,131,132,133,134,135,136,137,138,139,140,141,142,143,144,145,146,147,148,149,150,151,152,153,154,155,156,157,158,159,160,161,162,163,164,165,166,167,168,169,170,171,172,173,174,175,176,177,178,179,180,181,182,183,184,185,186,187,188,189,190,191,192,193,194,195,196,197], the limited number of references in the aforementioned reviews (between 38 [13] and 93 [16]) indicate that these reviews are either outdated or incomplete.

The aim of this study is to provide clinicians, basic science researchers and other stakeholders in healthcare with a comprehensive overview of what is known today regarding the effects of the exposure of musculoskeletal tissue to ESWs. This should help to further understand this fascinating, non-invasive treatment modality that is highly efficient and has a very good safety profile in the treatment of many pathologies of the musculoskeletal system. Because of the variety of different tissues that make up the musculoskeletal system, as well as of the different motivations for performing ESWT (ranging from pain relief to tissue regeneration), we divided our review into three areas focusing on bone and cartilage, connective tissue and muscle/nerve tissue.

## 2. Materials and Methods

PubMed and Web of Science were searched for “shock wave OR shock waves OR shockwave OR shockwaves NOT urol* NOT stone NOT review NOT clinical trial” from the days of inception of these databases until 30 September 2021, according to the PRISMA (Preferred Reporting Items for Systematic Reviews and Meta-Analyses) [198] guidelines. Duplicates were excluded.

For each identified publication, it was determined by reading the title and abstract whether the publication represented a study on the effects of exposure of musculoskeletal tissue to extracorporeal shock waves; the studies only addressing the treatment of skin with ESWT were excluded. All this was independently undertaken by T.W. and C.S. The results were compared and discussed until an agreement was achieved.

Subsequently, all the selected studies were classified with regard to the type of tissue (bone and cartilage, connective tissue or muscle/nerve tissue, respectively) that was exposed to ESWs. Furthermore, it was determined for each selected study whether (i) morphological, functional and radiological findings, (ii) findings of molecular biological investigations and/or (iii) findings of histological investigations were reported. All this was independently undertaken by T.W. and L.J., and the results were compared and discussed until an agreement was achieved.

**Figure 1:**
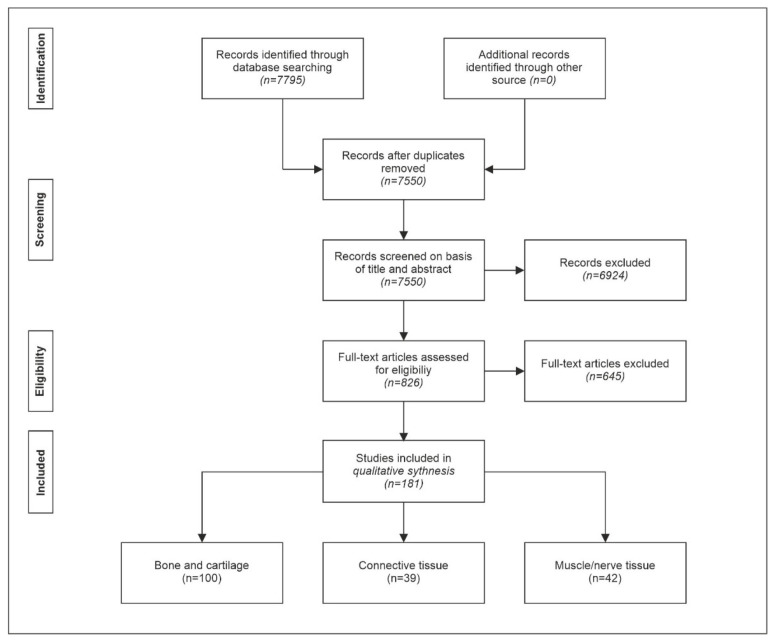
Systematic review flowchart of the literature search regarding studies on the effects of exposure of musculoskeletal tissue to extracorporeal shock waves performed according to the PRISMA guidelines [198] on 30 September 2021.

The strategy of the literature search is summarized in Figure 1.

## 3. Results

The results of this systematic review are summarized in Table 1, Table 2 and Table 3, with a distinction being made between effects of the exposure of bone and cartilage tissue (Table 1), connective tissue (Table 2) and muscle and nerve tissue (Table 3) to ESWs. Within each table, the results are arranged chronologically, with the most recent findings presented first. More details of the studies listed in Table 1, Table 2 and Table 3 are provided in Tables S1–S3.

### 3.1. Effects of the Exposure of Bone and Cartilage Tissue to Extracorporeal Shock Waves

Our systematic review revealed 100 studies that addressed the effects of ESWs on bone and cartilage tissue [6,18,19,20,21,22,23,24,25,26,27,28,29,30,31,32,33,34,35,36,37,38,39,40,41,42,43,44,45,46,47,48,49,50,51,52,53,54,55,56,57,58,59,60,61,62,63,64,65,66,67,68,69,70,71,72,73,74,75,76,77,78,79,80,81,82,83,84,85,86,87,88,89,90,91,92,93,94,95,96,97,98,99,100,101,102,103,104,105,106,107,108,109,110,111,112,113,114,115,116]. These studies were published between 1988 and 2021, with 51 (51%) of these studies published during the last ten years (2012–2021). Eighty-five of these studies (85%) applied fESWs, eleven (11%) of these studies applied rESWs, two (2%) of these studies applied both fESWs and rESWs, and in two (2%) of these studies it was not described whether fESWs or rESWs were applied. The majority of these studies (64 of 100, i.e., 64%) described animal experiments; primary or secondary cell-culture experiments were described in 23 (23%) or 7 (7%) of these studies, respectively. Three (3%) of these studies combined animal experiments with primary cell-culture experiments; one (1%) of these studies combined animal experiments with secondary cell-culture experiments, and two (2%) of these studies were conducted ex vivo without animal experiments and cell-culture experiments (details are provided in Table S1). Very different effects of ESWs on bone and cartilage tissue were addressed in these 100 studies; these effects are summarized in Table 1.

### 3.2. Effects of Exposure of Connective Tissue to Extracorporeal Shock Waves

Our systematic review revealed 39 studies that addressed effects of ESWs on connective tissue [117,118,119,120,121,122,123,124,125,126,127,128,129,130,131,132,133,134,135,136,137,138,139,140,141,142,143,144,145,146,147,148,149,150,151,152,153,154,155]. These studies were published between 1994 and 2021, with 18 (46.2%) of these studies published during the last ten years (2012–2021). Thirty (76.9%) of these study applied fESWs, and nine (23.1%) of these studies applied rESWs. The majority of these studies (24, i.e., 61.5%) described animal experiments; primary or secondary cell-culture experiments were described in nine (23.1%) or three (7.7%) of these studies, respectively. One study each (2.6% each) described cell-culture experiments (not further specified), experiments on fertilized chicken embryos and a human experiment (details are provided in Table S2). As in case of the exposure of bone and cartilage tissue to ESWs (Table 1), very different effects of ESWs on connective tissue were addressed in these 39 studies. These effects are summarized in Table 2.

### 3.3. Effects of Exposure of Muscle and Nerve Tissue to Extracorporeal Shock Waves

Our systematic review revealed 42 studies that addressed effects of ESWs on muscle and nerve tissue [156,157,158,159,160,161,162,163,164,165,166,167,168,169,170,171,172,173,174,175,176,177,178,179,180,181,182,183,184,185,186,187,188,189,190,191,192,193,194,195,196,197]. These studies were published between 1998 and 2021, with 25 (59.5%) of these studies published during the last ten years (2012–2021). Twenty-eight (66.7%) of these study applied fESWs, 10 (23.8%) of these studies applied rESWs, and in four (9.5%) of these studies it was not described whether fESWs or rESWs were applied. The vast majority (39, i.e., 92.9%) of these studies described animal experiments; two (4.8%) of these studies described primary cell-culture experiments, and one (2.4%) of these studies combined animal experiments with primary cell-culture experiments (details are provided in Table S3). As in case of the exposure of bone, cartilage tissue and connective tissue to ESWs (Table 1 and Table 2), very different effects of ESWs on muscle and nerve tissue were addressed in these 42 studies. These effects are summarized in Table 3.

## 4. Discussion

Based on the results summarized in Table 1, Table 2 and Table 3, we established ten take-home messages regarding the effects of exposure of musculoskeletal tissue to extracorporeal shock waves. These take-home messages are summarized in Table 4 and discussed below.

The first take-home message of this study is that compared to the effects of many other forms of therapy; the clinical benefit of extracorporeal shock wave therapy does not appear to be based on a single mechanism. Most of the basic studies on medical therapies run exactly opposite to the studies on the mode of action of ESWT. In preclinical research, mechanisms are often sought that are later clinically tested for their benefit. However, for the treatment indications of ESWT on the musculoskeletal system, mainly the clinical success is known to date, while, in contrast, the molecular and cellular causes for this success are widely unknown. Thus, studies of the mode of action of ESWT are based on rational considerations of the mechanisms by which clinical success might occur. In the numerous studies, a variety of effects were described, most of which are desirable for the respective indication. Many of these mechanisms are not causally related, so that it is obvious that the combination of different effects leads to the therapeutic success of ESWT.

The second take-home message of this study is that different tissues respond to the same mechanical stimulus in different ways. Based on many years of clinical experience and numerous clinical studies, various pathologies of the musculoskeletal system are known nowadays that can be successfully treated with ESWT [8,9,10,11]. These indications include mainly degenerations and injuries of muscle, bone and cartilage tissue. From basic research, a wide variety of effects at the molecular and cellular levels were described to date, whereby the effects of the ESWs differ in each case from the tissue treated. On the one hand, very tissue-specific reactions were observed. For example, while the enhancement of the osseous differentiation of stem cells occurred in the bone [23,48], the differentiation of stem cells into the osteocytic lineage was not observed in tendon tissue [125]. On the other hand, there are similar effects that were observed, despite the different tissues, such as an increase in the expression of vascular endothelial growth factor (VEGF) after exposure to ESWs in the bone and cartilage tissue [42,58,69,73,90,97], nerve tissue [170,175] and connective tissue [137]. This leads to the conclusion that ESWs generally promote angiogenesis, despite the fact that some studies described no effects after the exposure of tissue to ESWs on the expression of VEGF [118,119,162], or even the reduced expression of VEGF [26]. In addition, the condition of the treated tissue also seems to play a role. For example, healthy tenocytes responded to the exposure to ESWs with a different protein expression pattern than tenocytes from tendinopathic or ruptured tendon tissues [134,141]. This highlights one of the key problems in evaluating studies of the effects of ESWs on the musculoskeletal system: due to differences in design and the prevailing conditions in these studies, comparisons are sometimes difficult to make.

The third take-home message of this study is that just because a mechanism of action of extracorporeal shock wave therapy was described in a study does not automatically mean that this mechanism was relevant to the observed clinical effect. Some of the many effects described include effects that, considered in isolation, would not be desirable for the success of the therapy. However, as clinically a treatment success is mostly shown, other mechanisms must play a greater role for the effect of ESWT. One example is the increased vascularization of tendon tissue after exposure to ESWs [97,133]; although increased vascularization is usually associated with tendon inflammation [199], clinical findings were shown to improve after treatment [148]. Likewise, in the treatment of muscular spasticity by ESWT, it is unlikely that a stimulating effect of ESWs on, for example, stem cells, has anything to do with the reduced muscle tone after ESWT (e.g., [200]). Thus, when deducing the modes of action of ESWT in certain pathologies of the musculoskeletal system, one should always relate certain modes of action to the pathology under investigation in order to not obtain incorrect conclusions.

The fourth take-home message of this study is that focused and radial extracorporeal shock wave therapy seem to act in a similar way. Numerous effects were described for both fESWT and rESWT, however, more effects were described for fESWT (Table 1, Table 2 and Table 3). This may be due to the fact that fESWT was developed before rESWT [10]. From a physics point of view, these two forms of ESWT appear to differ greatly. Focused ESWs are generated by three methods that are named electrohydraulic, electromagnetic and piezoelectric [10]. Additionally, unlike rESWs, fESWs are generated in water that is inside the applicator [201]. In contrast, rESWs are generated by the acceleration of a projectile in a tube (through compressed air or a magnetic field), and the projectile hits an applicator at the end of the tube. Through contact with the skin via contact gel (to facilitate transmission), the rESWs are transmitted into the treated tissue [201]. As a result of these different mechanisms of ESW generation, rESWT has more of a superficial effect on tissues, while fESWT can also affect deeper tissues [10,201].

Some authors argued that rESWs should not be called shock waves, since they lack the characteristic physical features of true shock waves, including a short rise time in the amount of nanoseconds, a high peak pressure and non-linearity [202]. The physical definition of a “true” shock wave is as follows [203]: a high positive peak pressure (P_+_), sometimes more than 100 Megapascal (Mpa), but more often approximately 50 to 80 MPa; a fast initial rise in pressure (T_r_) during a period of less than 10 nanoseconds (ns); a low tensile amplitude (P_−_, up to 10 MPa); a short life cycle (I) of approximately 10 microseconds (μs); and a broad frequency spectrum, typically in the range of 16 Hertz (Hz) to 20 MHz. It is well-known that rESWs are not “true” shock waves in the strict physical sense outlined above [202]. This is because rESWs show a lower positive peak pressure (~10 MPa) and a substantially longer rise time (~600 ns), and have thus been termed radial pressure waves by some authors [204]. However, in 2007, it was already noticed that for treatment protocols at low-energy settings, neither piezoelectric nor electromagnetic fESWT devices generated true shock waves according to the physical criteria set out above [202]. With respect to the various ESWT devices’ abilities to generate shock waves as opposed to pressure waves, the initial concept can thus be refined into a concept that considers high-energy settings as a prerequisite for the generation of true shock waves. For clinical applications of ESWT, however, a more feasible concept of therapeutic shock wave technology needs to factor in two more considerations: that biological cells and tissues can differentiate between true shock waves and pressure waves, but cannot differentiate between radial or focused wave forms. As to the former point, it is certainly reasonable to differentiate between shock waves and pressure waves in terms of the differences in positive peak pressure delivered to the pathologic site. However, the question arises whether therapy success in many pathologies of the musculoskeletal system requires “true” shock waves [205]. It appears that this is not the case. With respect to the differentiation between rESWs and fESWs, under plain geometric considerations it is highly unlikely that tissues and cells can differentiate whether they are affected by focused or by radial acoustic waves—the only difference is in the number of affected cells. In consequence, it appears that, clinically, “a wave is a wave” regardless of whether it is generated with an fESWT device or a rESWT device. Much more important is whether sufficient ESWT energy is achieved where it is needed in the body.

Cavitation can be generated only during the shock wave’s tensile phase [206]. Of note, both fESWs and rESWs can generate vaporous cavitation [206]. Vaporous cavitation is assumed to play an important role in mediating molecular and cellular mechanisms of action of ESWT in biological tissues, presumably via the mechanical activation of membrane-bound signaling molecules, which, in turn, elicit cellular responses [206]. Yet, many questions remain open concerning the therapeutic effects of vaporous cavitation during ESWT. For example, it was found that tissues exposed to ESWs show a subsequent decrease in proinflammatory neuropeptides, similar to a “wash-out” effect [193]. This correlates well with the long-term analgesic effect mediated by ESWT in tendinopathies [10]. Yet, it remains unknown which effects vaporous cavitation has on the unmyelinated terminal endings of nociceptive fibers (i.e., C fibers) in the peripheral nervous system. More generally speaking, it is still unknown as to whether the therapeutic benefits of ESWT are due mainly to the positive (i.e., shear stress) or negative (i.e., cavitation) pressures, or a combination of both, in order to optimize treatment protocols [10]. Because of the potentially deleterious side effects of vaporous cavitation on the body, it is imperative to realize that both fESWT devices and rESWT devices can in fact generate vaporous cavitation in the treated tissue.

In summary, it is reasonable to hypothesize that further research into the effects of exposure of musculoskeletal tissue to fESWs and rESWs will demonstrate more similarities than dissimilarities between these modalities. Nevertheless, due to the differing energy distribution of both treatment forms in the target tissue, different energy-dependent effects may occur (e.g., [102]).

The fifth take-home message of this study is that extracorporeal shock wave therapy stimulates both progenitor and differentiated cells and has positive effects on the pathologies of bone and cartilage. A central aspect for the treatment of degenerations and injuries of muscles, tendons, bones and cartilage using ESWT is the activation of the respective tissue-specific cells. The mechanical pressure on the cells themselves leads to an increased expression of cell-specific proteins and cell viability. In bone, for example, there are several mechanisms by which bone growth is promoted and the activity of fully differentiated cells is increased. Numerous studies showed the upregulation of bone morphogenetic protein 2 (BMP-2) after the exposure of bones to fESWs [47,67,104]. BMP-2 plays a major role in osteoblast differentiation by transforming osteoblast precursor cells into mature osteoblasts that form healthy bones [207]. On the other hand, for proteins, such as RANKL, which, in turn, plays a role in osteoclast differentiation [208], a reduced expression was found after exposure to ESWs [19,63,80]. Furthermore, cavitation induced by ESWs can cause so-called “microcracks”, which is a stimulus for bone remodeling and new bone formation [209]. It was demonstrated in the bones of horses that fESWs can induce new microcracks, and rESWs can extend the length of existing microcracks [102]. When observing the effects of ESWT on the activity of different cell types, an increase in activity in tissue-specific cells, such as fibroblasts [68,124] and osteoblasts [39,83], but, at the same time, a reduced activity of osteoclasts [19], was observed. Together with the reduced RANKL expression, this could indicate a positive effect of ESWT on bone formation, as well as an improvement of diseases affecting the skeletal system, such as osteoporosis. In fact, ESWT shows positive effects in the treatment of these indications [8,210].

The sixth take-home message of this study is that extracorporeal shock wave therapy apparently mimics the effect of capsaicin by reducing substance-P concentration. In pathologies of tendons, muscle injuries and dysfunctions, as well as in osteoarthritis, the inflammatory cycle plays a crucial role, as does nociception for the quality of life of the patients. Substance P is a neuropeptide, which, once released after the activation of the TRPV1 receptor on mainly polymodal C-fibers [211], primarily activates the neurokinin-1 receptor (NK1R) [211,212]. Substance P plays an important role in nociception and neurogenic inflammation [213] through several intracellular pathways [212]. Therefore, in recent years, special attention was paid to capsaicin, a naturally occurring alkaloid that has certain reducing effects on substance-P concentration. Specifically, after application to the peripheral nerve, one of the effects of capsaicin was shown in an activation of the TRPV1 channel, mainly in the terminal endings of nociceptive fibers (especially C fibers), which initially does not lead to a reduction in pain and inflammation as an increase in substance-P concentration is to be expected [211,214]. By releasing substance P from the nerve fibers and simultaneously blocking the axoplasmic transport [215], the terminals are then depleted of their substance-P content [211,214]. However, whether this mechanism is (in addition to reducing inflammation [216]) also responsible for the pain relief with local capsaicin application is currently highly debated [217]. With ESWT, on the other hand, there is evidence that one of the analgesic effects is due to a reduction in the substance-P concentration in the tissue under treatment [191,193], thereby removing substance P from the C fibers. The mechanism behind this is probably a detrimental effect of ESWs on the TRPV1 channel. As with capsaicin, a similar time course of alterations in the amount of substance P in the periosteum was found after exposure of the femur of healthy rabbits to fESWs [191,193]. This may break the inflammatory cycle created by substance-P release, and thus has a different mechanism than medications, such as non-steroidal anti-inflammatory drugs (NSAIDs) that inhibit cyclooxygenase [218], but still helps reduce inflammation. In addition, both substance-P and calcitonin gene-related peptide expressions were demonstrated to be reduced in dorsal root ganglia after the exposure of peripheral tissue to ESWs [184,186,192]. The effect on the local inflammatory circuit is probably additionally enhanced by this. Due to the local application of ESWs, this effect is limited to the treatment region and the affected spinal cord segments, proven at least for substance P [184]. An important result of this is that ESWT does not induce the typical adverse events of treatments with NSAIDs, such as gastrointestinal ulcers and renal damage [218].

The seventh take-home message of this study is that extracorporeal shock wave therapy apparently mimics effects of injection of Botulinum toxin A by destroying endplates in the neuromuscular junction. Botulinum toxin A (BTX-A) injections are nowadays widely used for treating spasticity, which mainly affects individual muscle groups. Examples include spasticity induced by stroke [219], spinal cord injury [220] and infantile cerebral palsy [221]. The central problem in muscle spasticity is constant overexcitation at the neuromuscular endplate. BTX-A effectively prevents the formation of a stable SNARE complex by cleaving one of its associated proteins, SNAP-25. Since the SNARE complex is essential for acetylcholine release, a block of the skeletal cholinergic neuromuscular transmission occurs [222]. As reports of potentially serious side effects of BTX-A injections for treating spasticity continue to emerge [223,224] and long-term effects of this treatment modality remain to be established, the question of new treatment options arises. Extracorporeal shock wave therapy, similar to BTX-A injection, can transiently reduce excitatory transmission at the neuromuscular endplate. In this regard, it was shown in a rat model that the exposure of muscles to rESWs reduced the compound muscle action potential while maintaining the latency [157,168]. The key mechanism of ESWs, in contrast to BTX-A, is most likely the destruction of end plates in neuromuscular junctions, whereby the damage was confined to the postsynaptic membrane [168]. In a recent randomized controlled trial, it was found that BTX-A injection was not superior to rESWT in the treatment of plantar flexor muscle spasticity in patients with cerebral palsy [200].

The eighth take-home message of this study is that extracorporeal shock wave therapy apparently imitates certain mechanisms of action of neural therapy. Neural therapy is a treatment commonly used in Europe for pain relief. Its aim is to normalize the nervous system through targeted injections of local anesthetics [225]. Local anesthetics, such as the commonly used procaine, cause a blockade of the voltage-dependent sodium channels of nerve fibers [226]. This causes a reversible blockade of excitation conduction in nerve fibers, i.e., nociceptive afferents are shut down [226]. ESWT may have a similar principle of action in order to reduce pain conditions. Specifically, it was shown that, after the exposure of the femur to fESWs, a selective destruction and decreased number of unmyelinated nerve fibers in the sciatic nerve of rabbits was induced [183]. C fibers, for example, as part of the nociceptive system, belong to the unmyelinated nerve fibers. Furthermore, ESWs were shown to induce disturbed integrity of myelin sheaths combined with reduced nerve conduction velocities in palmar digital nerves in horses [190], as well as a reduced number of epidermal nerve fibers in the skin [189]. In summary, these results suggest that ESWT can reduce peripheral nerve function and conduction, without affecting the performance of professional athletes [227]. This mechanism may be central to the reduction in pain perception following ESWT, given the possibility that the transmission of nociceptive signals via peripheral nerves is impaired. Furthermore, it cannot be excluded that ESWT influences the conduction ability of sensitive nerves through the activation of gate-control mechanisms in the spinal cord [228]. Compared to neural therapy, a recent study demonstrated that, in patients with myofascial trigger points in the upper trapezius, both the repeated injection of 1% lidocaine and rESWT resulted in reduced pain alongside improved muscle elasticity, pressure pain threshold and neck disability index [229].

The ninth take-home message is that extracorporeal shock wave therapy apparently imitates certain mechanisms of manual therapy treatments. Many manual therapy treatments, such as massages, are aimed at achieving effects, including improved blood circulation, angiogenesis and reduced lymph congestion [230]. These effects were also observed after ESWT. For example, the exposure of skin and muscle tissue to both fESWs and rESWs resulted in a significant increase in the local microcirculation [126,131,173]. A positive effect of ESWT was also described on lymphatic drainage [137], and increased angiogenesis after exposure to ESWs was found in both blood vessels [131,165] and lymph vessels [137]. In addition, ESWT has a stimulating effect on the expression of lubricin in fasciae and tendon sheaths [135]. Lubricin was shown to induce an improvement in tendon gliding in vivo, and the absence of lubricin was demonstrated to significantly limit tendon mobility [231]. Of note, tendon gliding plays a major role in the rehabilitation of tendinopathies and tendon injuries [232]. Furthermore, rESWT was shown to significantly improve immobility-related muscle contractures and muscle fibrosis [156] in a rabbit model. A possible mechanism behind this is the reduced collagen deposition that was observed after treatment. However, it is unclear whether ESWT can also improve fascial fibrosis. As this is an alteration within the collagen fiber layers due to large amounts of undirected collagen material deposition [233,234], ESWT could also have a positive effect here.

The tenth take-home message is that even the most sophisticated research into the effects of exposure of musculoskeletal tissue to extracorporeal shock waves cannot substitute clinical research in order to determine the optimum intensity, treatment frequency and localization of extracorporeal shock wave therapy. Since this study was mainly about the different mechanisms of ESWT, no optimal treatment settings can be determined from the results summarized in Table 1, Table 2 and Table 3. In several studies, certain processes at the cellular level were described at certain points in time, which even contradicted each other in part. For example, while the exposure of cells to ESWs often led to reduced cell viability shortly after exposure, an increase in cell viability was observed in the further course of observation [84]. Therefore, it is reasonable to hypothesize that some biological changes only occur at a certain time, which, however, must be carefully considered in the study protocol and the measurements. In addition, some effects of the exposure of cells and tissue to ESWs were found only at certain energy levels [105,157] and numbers of applied ESWs [42]. Some studies even showed that the exposure of musculoskeletal tissue with ESWs with increasing EFD did not necessarily lead to better outcomes [105,106]. In summary, the only way to further optimize clinical application of ESWT is to perform more and better clinical research on this fascinating treatment modality. It is obvious that the results of basic research may be inspirational in this regard.

This systematic review had three limitations. First, only PubMed and Web of Science were searched. However, considering the fact that, in this review, considerably more studies were considered than in previous reviews on the same topic [13,14,15,16,17], it is reasonable to hypothesize that, in the present investigation, the risk to overlook any relevant study on the effects of exposure of musculoskeletal tissue to extracorporeal shock waves was minimized. Second, no meta-analysis of the presented data was performed. However, as outlined, particularly in the take-home messages 1–3 and 5, this appears to not be possible. Third, this review did not address all the potential indications of ESWT, but was restricted to musculoskeletal tissue. The mechanisms of action of ESWs in the treatment of, e.g., acute and chronic soft tissue wounds (e.g., [235]) or coronary artery disease (e.g., [236]) with ESWT may or may not be the same as discussed in this investigation.

## 5. Conclusions

The complementary effects of ESWT in the treatment of musculoskeletal pathologies make it an effective form of therapy that can be used alone or in combination with other therapeutic modalities. Not to be underestimated is the possibility of using ESWT as a supportive measure for any myofascial imbalances and functional movement restrictions underlying the pathologies. This is explained by the effects of ESWT on the myofascial units, such as the reduction in muscle tone, the decreased inflammatory activity and the effect on trigger points. Further studies, especially clinical studies, are needed for the future use of ESWT. To date, there is still minimal evidence on the ideal treatment settings, intensity, duration, localization and applied energy to provide the best possible treatment.

## Figures and Tables

**Table 1:** Effects of the exposure of bone and cartilage tissue to extracorporeal shock waves (more details of the studies listed in this table are provided in Table S1).

Ref.	First Author	Year	M	Morphological, Functional and Radiological Findings		
					Findings of Molecular Biological Investigations	
						Findings of Histological Investigations
[18]	Li	2021	F	Increased mineral apposition rates, trabecular bone volume, number, thickness; decreased trabecular separation		
					Increased expressions of ALP, OCN, RUNX2, OPG, SMAD2	
[19]	Inoue	2021	R	Increased trabecular bone microarchitecture and bone strength		
					Decreased RANKL	
[20]	Inoue	2021	R	Increased bone/tissue volumes		
						Increased osteoblast surface, decreased number of sclerostin-positive osteocytes
[21]	Zhao	2021	R		Unaltered expressions of OCN, RUNX2, COL2, SOX9; decreased expressions of CEBPα and PPARγ; increased expression of YAP	
						Increased proliferation
[22]	Kobayashi	2020	F	Increased bone union rate, radiographic score		
						Increased enchondral ossification, chondrogenic differentiation without inhibited proliferation
[23]	Alshihri	2020	F			Unaltered cell migration; increased proliferation and osteogenic differentiation
[24]	Hsu	2020	F	Increased bone strength, bone mineral density, trabecular thickness, bone /tissue volumes, porosity		
						Increased expressions of BMP2, BMP4 and Wnt3a signaling; unaltered expression of IGF1
[25]	Ramesh	2020	R	Increased bone length		
						Increased number of proliferative chondrocytes of growth plate’s cartilage and diameter of hypertrophic chondrocytes; activation of IGF1 and NFkb; increased levels of BCL2 and BCL-xL
[26]	Colbath	2020	F		Increased expression of ALP, decreased expressions of TGFb and VEGF	
[27]	Hashimoto	2019	F		Increased expressions of COL2a1, ACAN, CCN2, SOX9	
						Increased meniscal healing score and BrdU/CCN2 ratio
[28]	Senel	2019	F	Bone mineral density, bone mineral content		
[29]	Kim	2019	F	Increased structure and bone quality		
					Decreased expressions of TNFa, IL1b, IL6, MMP3, MMP13, BMP7	
						Increased cell viability; decreased number of apoptotic cells and pro-inflammatory, cartilage degradation markers
[30]	Buarque de Gusmao	2019	F/R		F: increased Akt and FAK activity and TGFb1 expression R: increased FAK activity, decreased Akt expression	
[31]	Cheng	2019	F	Enhanced bone volume and trabecular thickness		
						Reduced synovitis and cartilage damage; decreased expression of MMP-13; enhanced expressions of RUNX2, SOX-9 and COL10A1; enhanced expressions of IGF1, TGFb1 and COL2 and decreased TUNEL activity
[32]	Ginini	2019	F	Increased mineral density, enhanced bone formation		
						Higher collagen orientation index, increased expressions of COL1 and OCN
[33]	Ginini	2018	F	Higher degree of bone formation and mature bone; increased bone mineral density, bone volume fraction, and trabecular thickness		
						Enhanced expressions of BMP2, VEGF and PCNA
[34]	Qi	2018	R	Improved International Cartilage Repair Society (ICRS) score and macroscopic osteochondral appearance		
[35]	Koolen	2018	F	Cortical screws: increased bone formation and screw fixation. Cancellous screws: no alterations		
[36]	Mackert	2017	F	Improved average stiffness and yield load		
					Increased expressions of COL1a1, NR3A1, IGF1, OCN, TRAP	
						Improved average ventral, dorsal and endosteal callus formation
[37]	Tan	2017	F		ESWT alone: increased levels of A2B receptors; ESWT in combination with adenosine and A2BR agonists downregulated ACAN, COL1A2, COL2A1, SOX9 and SOX6	
						ESWT + adenosine and A2BR agonists: inhibited chondrogenic differentiation
[38]	Hsu	2017	n.s.		Increased expressions of ERK1, OPG, ALP, MMP13; potential activation of the 1α,25-Dihydroxyvitamin D3 Rapid Membrane Signaling Pathway	
						Increased expression of PDIA3
[39]	Yilmaz	2017	F	Increased osteoblastic activity, improved pain score		
						Lower modified Mankin score
[40]	Wang	2017	F	Improved OARSI score and gross pathological changes, less cartilage defects, higher bone mineral density and bone volume, improved bone porosity and yield stress		
						Increased expressions PCNA and OCN, decreased expression of TUNEL
[41]	Chen	2017	F	In vivo: improved bone volume, trabecular volume, BV/TV, bone thickness and bone mineral density		
					In vitro: increased expressions of COL1, RUNX2, OSX and ALP	
						In vitro: enhanced proliferation and osteogenic differentiation; in vivo: increased bone formation and expressions of RUNX2 and OSX
[42]	Onger	2017	F	500 impulses per treatment: unaltered bone volume/bone density 1000 impulses per treatment: enhanced bone volume/bone density		
						500 impulses per treatment: enhanced capillary volume, decreased connective tissue volume 1000 impulses per treatment: enhanced capillary volume; more positive areas of staining with VEGF, collagen antibody, BMP7 compared to control, but decreased capillary volume compared to 500 impulses; unaltered connective tissue volume
[43]	Wang	2017	F	Improved OARSI score and gross pathological changes, less cartilage defects, improved BV/TV ratio, improved bone porosity and trabecular thickness		
						Decreased expression of TUNEL; higher amount of PCNA-positive cells and increased vascular density; increased cartilage thickness and sectional cartilage area; decreased modified Mankin score
[44]	Lama	2017	F	Prevention of bone-weight reduction and trabecular microarchitecture deterioration; restored serum parameters of ALP, RANKL, OPG and PTH due to illness		
					Reduced cathepsin k, TNF-α levels, PPARγ and adiponectin transcription; increased RUNX2 and BMP2 expressions	
[45]	Catalano	2017	F		Increased ERK phosphorylation, ROS formation, RUNX2, ALP, BMP2	
[46]	Ma	2017	F	Higher bone volume per tissue volume, trabecular thickness, trabecular number, osteoblast surface/bone surface, osteoid surface/bone surface, osteoid thickness, mineralizing surface/bone surface, mineralizing apposition rate and bone formation rate as well as a reduced trabecular separation		
[47]	Huang	2016	F		Increased expressions of OPG and BMP-2	
[48]	Notarnicola	2016	F		Increased expressions of BMP, ALP, OCN, COL1A1 and RUNX2	
						Enhanced cell adhesion and proliferation
[49]	Zhai	2016	F		Increased expression of OCN, core-binding factor α1 and decreased PPARγ	
						Increased ALP content
[50]	Dias dos Santos	2015	F		Increased contents of sulfated glycosaminoglycans and hyaluronic acid	
[51]	Wang	2014	F	Reduced arthritic area of injury joint, enhanced bone mineral density and bone strength, improved subchondral plate thickness and bone porosity, reduced cartilage damage		
						Increased Mankin and Safranin O scores, improved alterations of the molecular levels due to the illness of Dickkopf-1, PCNA, VEGF and BMP-2
[52]	Muzio	2014	F		Decreased ALP and OCN	
						Increased cell growth
						Increased SMAD phosphorylation
[53]	Oktas	2014	F	No radiologic differences		
						Excised periosteum group: positive effect on bone healing
[54]	Sun	2013	F		Shockwave-dependent ATP release that activated P2X7 receptors and downstream signaling events, which induced the differentiation	
[55]	Suhr	2013	F			Extended growth rate, proliferation, migration, cell tracking and wound healing; ameliorated cell migration meditated by active remodeling of the actin cytoskeleton as indicated by increased directed stress fiber formations
[56]	Lyon	2013	F	Increased bony density		
						More mature bone formation, better healing, higher density of the cartilage
[57]	Wang	2013	F	Increased bone mineral density		
						Improved Mankin and Safranin O scores; increased COL2; decreased MMP13
[58]	Wang	2013	F			Treatment 1–2 times per week: improved Makin and Safranin O scores; increased COL2; decreased MMP13; increased vWF, VEGF, BMP-2 and osteocalcin; deteriorated effects after 3 treatments per week
[59]	van der Jagt	2013	F	Increased cortical volume (CtV), higher trabecular connectivity and more plate-like and thicker trabeculae, increased trabecular bone volume fraction		
[60]	Oztemur	2013	R	No changes in bone length		
						Increased blood vessel density, highly basophilic matrix and abundance of the differentiating chondrocytes
[61]	Gollwitzer	2013	R	New bone formation		
[62]	Altuntas	2012	R			Higher specimens’ mean scores in bone fracture healing
[63]	Notarnicola	2012	F		Reduction in COL1, OSX, bone sialoprotein and RANKL expressions, OCN and osteopontin; in summary: inhibiting effect on osteoclastogenesis	
[64]	Zhao	2012	R	Decreased NO level, and severity of cartilage lesions		
						Decreased chondrocyte apoptosis, enhanced Mankin score
[65]	Kearney	2012	F			Increased cambium cell number, cambium cell thickness, osseous tissue and callus area, larger amount of osteoprogenitor tissue; improved results in combination with a bioactive scaffold
[66]	Xu	2012	F		Promotion of Integrin alpha-5 and beta-1 expressions; induction of phosphorylation of FAK, which led to increased adhesion and migration of osteoblasts	
[67]	Wang	2012	F			Improved Mankin and Safranin O scores, increased COL2, VEGF, BMP2 and OCN expressions
[68]	Erturk	2012	F	No alterations in MRI		
						Edema, increased fibroblastic activity, neovascularization
[69]	Wang	2011	F	Increased BMD, bone strength, modulus of elasticity		
						Decreased Mankin score; improved Safranin O staining results; increased expressions of VWF, VEGF, BMP2, OCN and ALP; decreased expression of CTXII, cartilage oligomeric matrix protein
[70]	van der Jagt	2011	F	Increased 99mTc-MDP uptake, increased trabecular and cortical bone volume, higher bone stiffness; no alterations in microcrack analysis		
						Soft tissue damage, no periosteal damage, de novo bone with active osteoblasts and osteoids
[71]	Notarnicola	2011	F		Increased expressions of RUNX2, COL1, OCN, IGF1, IGFBP3; decreased expressions of IGFBP-4 and -5	
[72]	Hausdorf	2011	F		Increased basic fibroblast growth factor; no significant alterations in TGFb	
[73]	Wang	2011	F	Increased bone mineral content		
						Increased bone tissue; decreased fibrous tissue; increased expressions of VEGF, VWF, PCNA, OCN and BMP2; decreased expression of TUNEL
[74]	Mayer-Wagner	2010	F		Increased COL2A1 expression	
						Ultrastructural expansion of the rough-surfaced endoplasmatic reticulum, detachment of the cell membrane and necrotic chondrocytes; increased tenascin-C and Chitinase-3-like protein 1; no alterations in Mankin score
[75]	Muzio	2010	F		Increased expressions of ALP, COL1, BMP-4, OCN	
						Increased osteoblast activity as well as number and size of calcium deposits
[76]	Lai	2010	F	Treatment with 14kV: increased mineral density, biomechanical bone strength, intense osteoblastic cell recruitment, new bone formation		
						Treatment with 14kV: intense osteoblastic cell recruitment, new bone formation, neovascularization, increased PCNA, VEGF, BMP-2; opposite effects after treatment with 21kV
[77]	Qin	2010	F	Higher fraction of new bone		
						Increased VEGF expression in hypertrophic chondrocytes, promotion of regeneration of the fibrocartilage zone
[78]	van der Jagt	2009	F	Diminished bone loss, higher trabecular bone-volume fraction		
						No differences in mineralization or osteoid appearance
[79]	Iannone	2009	F		Increased expression of IL10; no alterations in TGFa, CD29 and CD105 expressions	
[80]	Tamma	2009	F		Increased expressions of BCL-2-associated X protein, RUNX2, OPN, bone sialoprotein, OCN and COL1; decreased RANKL/OPG ratio suggesting inhibition of osteoclastogenesis	
[81]	Lee	2009	F	Increased callus formation and both extension and flexion stiffness		
[82]	Tam	2009	F	Enhanced trabecular bone mineral density, trabecular bone-volume fraction, trabecular thickness		
						Increased mineral apposition rate
[83]	Hofmann	2008	F		Altered expression of several genes involved in bone formation, osteoblast differentiation and skeletal development; no alterations in RUNX2, OSX, osteopontin, osteonectin, OC, TGFb1 expressions	
						Enhanced mineralization and number of ALP-positive osteoblasts
[84]	Tam	2008	F		Decreased cell viability 6 days after treatment; increased viability 18 days after treatment; increased cell proliferation 18 days after treatment	
						Enhanced mineralization 35 days after treatment and AP activity 18 days after treatment
[85]	Lee	2008	F	New bone formation		
						Superior fusion mass
[86]	Wang	2008	F	Increased bone strength		
						Increased cortical bone formation; higher number of newly formed vessels; increased expression of VEGF, nitric oxide synthase 3, PCNA and BMP-2
[87]	Moretti	2008	F		Decreased expression of IL10 and TNFa in both groups; no alteration in b1-integrin expression	
[88]	Tischer	2008	F	Dose-dependent new bone formation		
						Dose-dependent new bone formation
[89]	Ozturk	2008	F			Increased epiphyseal plaque thickness and number of chondrocytes
[90]	Ma	2007	F		Increased VEGF expression	
						Increased bone and osteoblast number; increased VEGF expression and microvessel density
[91]	Murata	2007	R		Augmented uniform gene transfection and increased activity of vector-expressed genes	
[92]	Benson	2007	R		Decreased synthesis of GAG; no alterations in NO or Prostaglandin E2 synthesis	
[93]	Martini	2006	F		Dose- and device-dependent cell viability and expression of ALP, Capicua Transcriptional Repressor Pseudogene, OCN and TGFb	
[94]	Bulut	2006	F	Increased callus volume		
						Advanced bone healing
[95]	Martini	2005	F	Enhanced transmembrane current and voltage dependence of Ca-activated/K channels		
[96]	Saisu	2005	F	Increased breadth of the acetabular roof and transient woven bone formation on the lateral margin		
[97]	Chen	2004	F		Increased TGFb1 and VEGF-A expressions	
						Increased cell density and cell number of RP59-positive mesenchymal stem cells, subsequently enhanced differentiation into chondrocytes and osteocytes
[98]	Saisu	2004	F	Enhanced bone mineral content, long-bone length and width		
[99]	Chen	2004	F		Increased ALPase, COL1, COL2 and OCN expressions and [3H]-thymidine uptake; increased expressions and phosphorylations of ERK and p38	
						Activated ERK and p38 expressions
[100]	Pauwels	2004	F	No alterations in bone elasticity		
[101]	Wang	2004	n.s.		Induced superoxide production; enhanced TGFb1, RUNX2, OCN and COL1 expressions; increased bone alkaline phosphatase activity	
						Increase in bone nodule formations, promotion of the CFU-stroma formation but not CFU-mix formation
[102]	da Costa Gomez	2004	F/R			R: increased microcrack length, fESWT: increased microcrack density
[103]	Takahashi	2004	F	Increased cortical thickening, bone mineral density, bone mineral content		
						Enhanced expressions of COL1A1, COL2A1, OC and OPN; no alterations in expression of COL10A1
[104]	Chen	2003	F	Increased callus size and calcium content, bone mineral density		
					Increased ALP activity, OCN production, PCNA, TGFb1 and BMP-2 expressions	
						Increased bone-tissue formation, progressive mesenchymal aggregation, enchondral ossification and hard callus formation
[105]	Martini	2003	F		High intensity treatment (28 kV): decreased viability; reduced cell respiration; depressed ALP and NO synthesis; decreased expressions of OCN, TGFb and Procollagen-type I carboxy-terminal propeptide (PICP); low intensity treatment (14 kV) showed contrary effects with increased viability and cell respiration, increased ALP and NO synthesis as well as OCN and PICP expressions; generally negative affection of PICP production	
[106]	Martini	2003	F		Increased NO, OCN and TGFb1 production after low energy application (14kV); decreased cell viability and expression of all examined proteins at high application intensities (28 kV)	
[107]	Dorotka	2003	F		Increased cytotoxity in both chondrocytes and BMSCs at high application intensities (0.17mJ/mm2), compared to lower energy levels and control; unaltered cell proliferation at all energy levels	
[108]	Wang	2003	F		Increased expressions of BMP2, BMP3, BMP4 and BMP7	
						Intensive mesenchymal cell aggregation, hypertrophic chondrogenesis and endochondral/intramembrane ossification; increased levels of PCNA, BMP2, BMP3 and BMP4
[109]	Maier	2002	F	Decreased bone metabolism after 10 days (detected by scintigraphy), but increased metabolism after 28 days; signs of soft-tissue oedema, epiperiosteal fluid and bone marrow oedema on MRI		
						Epiperiostal deposits of hemosiderin
[110]	Wang	2002	F		Increased ALP activity and TGFb1 expression	
						Promotion of bone marrow stromal, but not hematopoietic cell growth; dose-dependent effect on formation of CFU osteoprogenitors
[111]	Wang	2001	F		Induction of cell membrane hyperpolarization and consecutive Ras activation; induction of RUNX2; increased activity of bone ALP; increased expressions of OCN and COL1	
						Increased bone-nodule formations
[112]	Wang	2001	F	More callus formations		
						More cortical bone and thicker, denser and heavier bone tissues
[113]	Vaterlein	2000	F	Neither macroscopic nor radiological alterations after high-intensity treatments		
						No histological alterations after high-intensity treatments
[114]	Peters	1998	F			Several damages to tissues after low-intensity treatment
[115]	Augat	1995	F	Neither alterations in biomechanical outcomes nor altered radiological results; tendency to deterioration of facture healing with increasing application intensities		
[116]	Forriol	1994	F	No effect on the periosteal surface of mature cortical bone, but on the endosteal surface induction of some new trabecular bone; delayed bone healing		
[6]	Graff	1988	F	Soft-tissue bleeding		
						Bone marrow hemorrhage and osteocyte damage 48 h after ESWT; increased callus and bone formation, focal regeneration, apposition of new bone, bone remodeling

Abbreviations: ACAN, aggrecan; Akt, protein kinase B; ALP, alkaline phosphatase; ATP, adenosine triphosphate; BCL, B-cell lymphoma; BMP, bone morphogenetic protein; BMSCs, bone marrow mesenchymal stem cells; BrdU, bromodeoxyuridine; CCN2, connective tissue growth factor; CEBPα, CAAT/enhancer binding protein; CFU, colony forming unit; COL, collagen; CTXII, C-telopeptide of collagen alpha-1(II) chain; ERK, extracellular signal-regulated kinases; F, focused extracorporeal shock waves; FAK, focal adhesion kinase; GAG, glycosaminoglycans; IGF, insulin-like growth factor; IL, interleukin; MMP, matrix metalloproteinase; NFkb, nuclear factor kappa-light-chain-enhancer of activated B cells; NO, nitric oxide; ns, not specified; NR3A1, estrogen-receptor alpha; OCN, osteocalcin; OPG, osteoprotegerin; OSX, osterix; PCNA, proliferating cell nuclear antigen; PDIA, protein disulfide-isomerase A; PPARγ, peroxisome proliferator-activated receptor gamma; PTH, parathyroid hormone; R, radial extracorporeal shock waves; RANKL, receptor activator of nuclear factor kappa-Β ligand; Ref, reference; ROS, reactive oxygen species; RUNX2, runt-related transcription factor 2; SMAD2, mothers against decapentaplegic homolog 2; T, type of extracorporeal shock waves; TGF, transforming growth factor; TNF, tumor necrosis factor; TRAP, tartrate-resistant acid phosphatase; TUNEL, terminal deoxynucleotidyl transferase dUTP nick end labeling; VEGF, vascular endothelial growth factor; vWF, von Willebrand factor; YAP, yes-associated protein.

**Table 2:** Effects of the exposure of connective tissue to extracorporeal shock waves (more details of the studies listed in this table are provided in Table S2).

Ref.	First Author	Year	M	Morphological, Functional and Radiological Findings		
					Findings of Molecular Biological Examinations	
						Findings of Histological Examinations
[117]	Haberal	2021	R			Decreased epidural fibrosis; unaltered acute/chronic inflammation and vascular proliferation
[118]	Heimes	2020	R		Increased expression of MMP-9; decreased expression of MMP-13; unaltered expression of inducible nitric oxide synthase 2, HIF1α, VEGF	
						Increased coverage of the transplant by vasculature, percentage of the vascularized area, increase in the vascularized area and number of vessel junctions
[119]	Lu	2020	F		Increased ACL remnant cell viability; BMSC: increased expressions of Ki67, COL1 and COL3; unaltered expressions of TGFb and VEGF	
						ACL cells: increased expression of COL1A1, TGFb and VEGF; BMSC: increased migration and expression of 5-Ethynyl-2’-deoxyuridine, COL1 and COL3; unaltered expression of VEGF and TGFb
[120]	Basoli	2020	F		Increased proliferation, ATP release, ROS production, expressions of IL8, MCP1, HSP90 and HSP27; unaltered expression of IL6	
[121]	Schnurrer-Luke-Vrbanić	2018	R			Higher multiplication of collagen fibers; faster organization of muscle fibers and vascularization by treatment with radial shockwaves
[122]	Cui	2018	F		Decreased expression of TGFb, a-SMA, vimentin, COL1A1, N-CAD and twist; increased expression of DNA-binding protein inhibitor ID1/2, E-CAD and FN after 24 h, but decreased expression of FN after 72 h	
						Decreased cell migration
[123]	Cai	2016	F		Initially decreased expression of IL6, IL8, MCP1 and TNFa; after 4 and more hours: increased expression of IL6 and IL8, unaltered expression of MCP1 and TNFa	
[124]	Hoch-strasser	2016	R		Induced mechanical cell destruction, dose-dependent decreased cell viability, increased growth potential of fibroblasts (not of JEG-3 cells), shift in proportion from G0/G1 to G2/M phase in fibroblasts (not in JEG-3 cells)	
						Cellular detachments, holes in monolayers, disruption of actin filaments
[125]	Leone	2016	F		Increased expressions of COL2A, SOX9, ALP and PPARy; unaltered expressions of OCN and RUNX2	
						Increased expression of differentiation markers in cells grown in specific differentiation media
[126]	Kisch	2015	F	Increased capillary blood velocity; unaltered postcapillary venous filling pressure		
[127]	Waugh	2015	R		Increased expressions of IL6, IL8, MMP2 complex and ProMMP9; unaltered expressions of IL1b, IL2, IL4, IL10, IL12p70, IL17A, VEGF, interferon-γ, active MMP9, ProMMP2 and active MMP2	
[128]	de Girolamo	2014	F		Increased expressions of SCX, IL1b, IL6, IL10, TGFb and VEGF; unaltered expressions of MMP3, MMP13, COL1A1, COL3A1 and TNFa; reduced NO synthesis	
[129]	Chow	2014	F			Increased fibrocartilage area and thickness, proteoglycan deposition, expression of SOX9 and COLII and Vickers hardness; unaltered expression of COL1
[130]	Cinar	2013	R	Decreased load to failure		
						Decreased collagen fiber density
[131]	Contaldo	2012	R			Enhanced expressions of caspase-3, PCNA and eNOS; increase in functional angiogenetic density and total wound score
[132]	Chow	2012	F	Increased load to failure, new bone area and new bone volume		
						Increased fibrocartilage zone and ratio of bone forming
[133]	Yoo	2012	F			Increased fibrillary diameter, vascularity, fibroblast activity, lymphocyte and plasma cell infiltration, dense histocytes; transient disorganization of collagen fibers
[134]	Leone	2012	F		Ruptured tenocytes: decreased expressions of COL1 and SCX; unaltered COL3, tenomodulin, tenascin-C	
						Healthy tenocytes: increased cell proliferation and migration
[135]	Zhang	2011	F			Increased lubricine expression
[136]	Penteado	2011	F			Unaltered blood-vessel number
[137]	Kubo	2010	F	Reduced ear thickness		
					Increased expressions of VEGF-C and VEGF-R3	
						Increased density of lymphatic vessels
[138]	Sugioka	2010	R			Increased introduction of NFkb decoy-FITC, activation of NFkb; decreased activation of NFkb after pretreatment with ESW+NFkb decoy-FITC
[139]	Berta	2009	F		Decreased viability; increased expression of TGFb1; increase in COL1 and COL3 expressions after 6 days after a primary decreased expression	
[140]	Bosch	2009	F		Increased expressions of COL1 and MMP14; decreased expression of MMP3	
						Unaltered total collagen content, disorganization of normal collagen structure; decreased percentage of degraded collagen 6 weeks after treatment after an increase 3 h after treatment
[141]	Han	2009	F		Healthy: increased expression of IL1; unaltered expressions of MMP1, MMP2, MMP9, MMP13, IL6 and IL13 Diseased: decreased expressions of MMP1, MMP13 and IL6; unaltered expressions of MMP2, MMP9, IL1 and IL13	
						Decreased cell viability
[142]	Byron	2009	R	Radiographic scores, scintigraphic navicular pool phase, delayed-phase region of interest density ratios		
[143]	Chao	2008	F		Increased total collagen concentration, NO production, expressions of PCNA, COL1, COL3 and TGFb	
						Decreased cell viability; increased cell proliferation
[144]	Wang	2008	F	Increased new bone formation, bone mineral status, tensile load and strength		
						Increased remodeling/alignment of collagen fibers, thicker and mature regenerated fibrocartilage zone
[145]	Bosch	2007	F		Unaltered DNA content, 3 h after treatment: increased GAG, total protein synthesis; 6 weeks after treatment: decreased GAG, collagen synthesis, noncollagenous protein synthesis, total protein synthesis	
						Unaltered total collagen content, disorganization of normal collagen structure; decreased percentage of degraded collagen 6 weeks after treatment after an increase 3 h after treatment
[146]	Kersh	2006	F			Unaltered percentage lesion, percentage disruption and gray scale, external width, fibroblast and tenocyte number, increased capillary density
[147]	Wang	2005	F	Increased trabecular bone around the tendons and tensile strength of tendon/bone interface, better bone/tendon contact		
[148]	Chen	2004	F	Increased load to failure		
						Decreased edema, swelling, inflammatory cell infiltration; increased expressions of TGFb, IGF1, tenocyte proliferation, neovascularization and progressive tendon tissue regeneration
[149]	Orhan	2004	F	Higher force to rupture		
						Less adhesion formation, increased number of capillaries
[150]	Hsu	2004	F	Increased ultimate tensile load		
						Increased hydroxyproline concentration; decreased pyridinoline concentration; unaltered number of blast-like tenocytes (4 weeks); increased number of mature tenocytes (16 weeks)
[151]	Orhan	2004	F			Disorganization of collagen fibers
[152]	Wang	2003	F			Increased number of neo-vessels and expressions of eNOS, VEGF and PCNA
[153]	Maier	2002	F			Exposure of tendons with high intensity ESWT: increased staining affinity, nuclear and fibrillar appearance paratendon: increased thickness, edema, capillary density
[154]	Wang	2002	F			New capillary and muscularized vessels, newly appeared myofibroblasts; no alterations in bone matrix, bone vascularization and osteocyte activity
[155]	Johannes	1994	F			Decreased cell viability, no alterations in cell growth

Abbreviations: a-SMA, alpha smooth muscle actin; ACL, anterior cruciate ligament; ALP, alkaline phosphatase; ATP, adenosine triphosphate; BMSCs, bone marrow mesenchymal stem cells; COL, collagen; F, focused extracorporeal shock waves; FITC, fluorescein isothiocyanate; FN, fibronectin; GAG, glycosaminoglycans; HIF, hypoxia-inducible factor; HSP, heat shock protein; IGF, insulin-like growth factor; IL, interleukin; MCP, monocyte chemoattractant protein; MMP, matrix metalloproteinase; NFkb, nuclear factor kappa-light-chain-enhancer of activated B cells; NO, nitric oxide; OCN, osteocalcin; PCNA, proliferating cell nuclear antigen; PPARγ, peroxisome proliferator-activated receptor gamma; R, radial extracorporeal shock waves; Ref, reference; ROS, reactive oxygen species; RUNX2, runt-related transcription factor 2; SCX, scleraxis; T, type of extracorporeal shock waves; TGF, transforming growth factor; TNF, tumor necrosis factor; VEGF, vascular endothelial growth factor.

**Table 3:** Effects of the exposure of muscle and nerve tissue to extracorporeal shock waves (more details of the studies listed in this table are provided in Table S3).

Ref	First Author	Year	M	Morphological, Functional and Radiological Findings		
					Findings of Molecular Biological Examinations	
						Findings of Histological Examinations
[156]	Huang	2021	R	Decreased total contracture angle, muscle contracture angle		
					Decreased expressions of TGFb and HIF1a	
						Decreased proportion of collagen fiber area
[157]	Kenmoku	2021	R	Energy flux density- and total energy-dependent decrease in CMAP, unaltered CMAP latency		
[158]	Park	2020	F	Increased print width, print area		
					Tendential increased expression of myelin basic protein	
[159]	Matsuda	2020	F	Improved BBB locomotor function, increased withdrawal threshold, abbreviated latency of MEPs, no alterations in MEP amplitude		
					Increased expressions of BDNF and TRKB	
						Increased expression of BDNF, reduced myelin damage and oligodendrocyte loss, decreased axonal damage
[160]	Langendorf	2020	R		Increased expressions of MyoD and myosin	
						Initially higher amount of mononucleated cells; at day 7, newly formed muscle fibers with less MNCs; unaltered number of cells immunopositive for CD31
[161]	Sagir	2019	F	Decreased EMG amplitude, increased EMG latency, improved sciatic functional index		
						Decreased myelin thickness, axon area and number
[162]	Feichtinger	2019	F	Improved load-to-failure testing results, intensity measurements in functional gait analysis		
					Unaltered expressions of stromal cell-derived factor 1, TGFb1, TGFb3 and VEGFR2	
[163]	Yang	2019	n.s.	Improved mechanical paw withdrawal threshold and thermal paw withdrawal latency		
					Decreased TNFa, NFkb, MMP9, IL1b, NOX1, NOX2, NOX4, oxidized protein, cleaved caspase 3, cleaved PARP, γ-H2AX, (p)-p38, p-JNK, p-ERK1/2, Nav.1.3, Nav.1.8 and Nav.1.9	
[164]	Mattya-szovszky	2018	R		Dose-dependent increase in myogenic factor 5, MyoD, PAX7 and NCAM; downregulation of these proteins at double exposure of the highest energy flux density	
						Increased cell viability at low energy flux densities, no alterations at higher energy flux densities
[165]	Yin	2018	F	Increased angiogenesis, decreased serum myoglobin/creatine phosphokinase		
					Decreased NOX1, NOX2, cleaved caspase 3, cleaved PARP, TGFb, (p-)SMAD3, ICAM1, MMP9, TNFa, NFkb, chemokine (C-C motif) ligand 5, TLR2, TLR4, IL1b, cytosolic cytochrome C, γ-H2AX; increased Bcl-2, p-SMAD1/5, BMP-2, mitochondrial cytochrome C	
						Decreased muscle-damaged/fibrosis/collagen-deposition areas
[166]	Shin	2018	R		Increased expressions of DCX, SOX2, GAP43 and MAP2	
						Increased expressions of DCX, SOX2, GAP43 and MAP-2
[167]	Luh	2018	F	Enhanced amplitude and latency of sensory nerve action potentials in combination with EMLA, compared to single EMLA and ultrasound+EMLA application		
[168]	Kenmoku	2018	R	Decreased CMAP amplitude, unaltered CMAP latency		
						Irregular end plates, unchanged axon terminals and muscle fibers, increased mean interjunctional fold interval
[169]	Chen	2017	n.s.	Improved mechanical paw withdrawal threshold and thermal paw withdrawal latency		
					Decreased expressions of TNFa, NFkb, MMP9, IL1b, GFAP, ox42, NOX1, NOX2, NOX4, oxidized protein, γ-H2AX, cytosolic mitochondria, cleaved capase-3, PARP, p-P38, p-JNK, p-ERK1/2, Nav.1.3, Nav.1.8 and Nav.1.9	
						Decreased expressions of p-P38+, peripherin+ cells, P38+ and NF200+ cells
[170]	Yahata	2016	F	Improved BBB locomotor score, withdrawal latency, 50% withdrawal threshold		
						Increased expressions of VEGF, CD31, a-SMA and 5-HT; increased area of spared white matter; decreased number of TUNEL-positive cells
[171]	Schuh	2016	F		Increased cell yield, BrdU assays, population doublings, S100b, c-Jun, GFAP and P75 expression; decreased P0 and P16 expressions, increased extracellular ATP levels immediately after application	
[172]	Lee	2016	n.s.	Decreased knee-joint angle		
[173]	Kisch	2016	F	Increased muscular blood flow		
[174]	Lee	2015	n.s.	Increased ankle angles (toe off + foot contact), improved sciatic functional index		
					Increased expression of NT3	
[175]	Yamaya	2014	F	Improved BBB locomotor score		
					Increased expressions of VEGF and VEGF-receptor 1	
						Increased NeuN-positive cells, VEGF staining
[176]	Fu	2014	F	Improved mechanical withdrawal threshold, thermal withdrawal latency		
[177]	Ishikawa	2013	R	Transfection of POMC gene		
[178]	Mense	2013	F	Decreased pressure pain threshold, improved locomotor activity		
						Increased number of PGP 9.5-IR nerve fibers
[179]	Hausner	2012	F	Increased amplitude, CMAP area		
						Increased number of myelinated axons, unaltered number of endoneural vessels
[180]	Kenmoku	2012	R	Decreased amplitude, unaltered CMAP latency		
						Decreased number of acetylcholine receptors
[181]	Yamashita	2009	R	Decreased mechanical allodynia		
						Increased ratio of β-endorphin-IR muscle cells and number of β-endorphin-IR muscle fibers; decreased number of CGRP-IR DRG neurons
[182]	Wu	2008	F	Decreased motor nerve conduction velocity; unaltered sciatic functional index and withdrawal reflex latency		
						Damage to the myelin sheath of large-diameter myelinated fibers
[183]	Hausdorf	2008	F			Decreased number of unmyelinated nerve fibers of femoral nerve; unaltered number of unmyelinated nerve fibers of sciatic nerve; unaltered size, number and myelin sheet of myelinated nerve fibers
[184]	Hausdorf	2008	F			Decreased number of neurons immunoreactive for substance P
[185]	Lee	2007	F	No changes in motor and vegetative functions		
						Decreased number of neurons during high-intensity treatment, dose-dependent myelin damage
[186]	Ochiai	2007	F	Increased walking duration		
						Decreased ratio of CGRP-positive dorsal root ganglion neurons
[187]	Wu	2007	F	Decreased motor nerve conduction velocity, unaltered sciatic functional index		
[188]	Murata	2006	F			Increased number of ATF3 and ATF-3/GAP-43 dual-IR neurons
[189]	Takahashi	2006	F			Decreased number of epidermal nerve fibers
[190]	Bolt	2004	R	Decreased sensory nerve conduction velocity		
						Disruption of myelin sheet
[191]	Hausdorf	2004	F		Increased substance-P release 6 and 24 h after treatment, decreased substance-P release 6 weeks after treatment; unaltered prostaglandin-E2 release	
[192]	Takahashi	2003	F			Decreased percentage of CGRP-immunoreactive dorsal root ganglion neurons
[193]	Maier	2003	F			Increased substance-P release after 6 and 24 h; decreased SP release after 6 weeks; no alterations in prostaglandin-E2 release
[194]	Haake	2002	F		Unaltered c Fos expression	
						Unaltered c Fos expression
[195]	Ohtori	2001	F			Decreased number of nerve fibers immunoreactive for PGP 9.5 and CGRP
[196]	Haake	2001	F			Unaltered expressions of met-enkephalin and dynorphin
[197]	Rompe	1998	F			Vacuolic swelling of axons, no disruption of nerve’s continuity

Abbreviations: a-SMA, alpha smooth muscle actin; ATF, activating transcription factor; ATP, adenosine triphosphate; BCL, B-cell lymphoma; BDNF, brain-derived neurotrophic factor; BMP, bone morphogenetic protein; BrdU, bromodeoxyuridine; CFU, colony forming unit; CGRP, calcitonin gene-related peptide; CMAP, compound muscle action potential; DCX, doublecortin; DRG, dorsal root ganglion; EMG, electromyography; EMLA, eutectic mixture of local anesthetics; ERK, extracellular signal-regulated kinases; Ff, focused extracorporeal shock waves; GAG, glycosaminoglycans; GAP, growth associated protein; GFAP, glial fibrillary acidic protein; HIF, hypoxia-inducible factor; ICAM, intercellular adhesion molecule; IL, interleukin; IR, immunoreactive; JNK, jun N-terminal kinases; MAP, microtubule-associated protein; MEP, motor evoked potentials; MMP, matrix metalloproteinase; MNC, mononucleated cells; MyoD, myoblast determination protein 1; Nav, sodium channel, voltage-gated; NCAM, neural cell adhesion molecule; NeuN, hexaribonucleotide binding protein-3; NFkb, nuclear factor kappa-light-chain-enhancer of activated B cells; NOX, NADPH oxidase; NT, neurotrophin; PARP, poly (ADP-ribose) polymerase; PAX, paired box protein; PGP, protein gene product; POMC, proopiomelanocortin; R, radial extracorporeal shock waves; Ref, reference; T, type of extracorporeal shock waves; TGF, transforming growth factor; TLR, Toll-like receptor; TNF, tumor necrosis factor; TRKB, tropomyosin receptor kinase B; TUNEL, terminal deoxynucleotidyl transferase dUTP nick end labeling; VEGF, vascular endothelial growth factor; 5-HT, serotonin.

**Table 4:** Take-home messages regarding the effects of exposure of musculoskeletal tissue to extracorporeal shock waves.

No.	Take-Home Message
1	Compared to the effects of many other forms of therapy, the clinical benefit of extracorporeal shock wave therapy does not appear to be based on a single mechanism.
2	Different tissues respond to the same mechanical stimulus in different ways.
3	Just because a mechanism of action of extracorporeal shock wave therapy was described in a study does not automatically mean that this mechanism was relevant to the observed clinical effect.
4	Focused and radial extracorporeal shock wave therapy seem to act in a similar way.
5	Extracorporeal shock wave therapy stimulates both progenitor and differentiated cells, and has positive effects on pathologies of bone and cartilage.
6	Extracorporeal shock wave therapy apparently mimics the effect of capsaicin by reducing substance-P concentration.
7	Extracorporeal shock wave therapy apparently mimics effects of injection of Botulinum toxin A by destroying endplates in the neuromuscular junction.
8	Extracorporeal shock wave therapy apparently imitates certain mechanisms of action of neural therapy.
9	Extracorporeal shock wave therapy apparently imitates certain mechanisms of manual therapy treatments.
10	Even the most sophisticated research into the effects of exposure of musculoskeletal tissue to extracorporeal shock waves cannot substitute clinical research in order to determine the optimum intensity, treatment frequency and localization of extracorporeal shock wave therapy.

## Data Availability

All relevant data are provided in the text.

## Supplementary Materials

The following supporting information can be downloaded at: https://www.mdpi.com/article/10.3390/biomedicines10051084/s1. Table S1: Details of the studies listed in Table 1. Table S2: details of the studies listed in Table 2. Table S3: Details of the studies listed in Table 3.
