# Supplementary material for: The Effects of the Exposure of Musculoskeletal Tissue to Extracorporeal Shock Waves
Source: Biomedicines. 2022 May 6;10(5):1084. doi: 10.3390/biomedicines10051084 (PMC9138291; doi:10.3390/biomedicines10051084)
Supplement: Supplementary file 1 [file biomedicines-10-01084-s001.zip › biomedicines-1713422-supplementary.pdf]

# Effects of exposure of musculoskeletal tissue to extracorporeal shock waves

Tobias Würfel, Christoph Schmitz and Leon L.J. Jokinen

## Supplementary Material

# Table S1: effects of the exposure of bone and cartilage tissue to extracorporeal shock waves

| Reference No. | Author | Year | Study type | Utilized device | Cell type investigated | Morphological, functional, radiological findings | Findings of molecularbiological examinations | Findings of histological examinations |
|---|---|---|---|---|---|---|---|---|
| [18] | Li et al. | 2021 | Primary cell culture + animal experiment | Epos Ultra, Dornier MedTech, Wessling, Germany | Rabbit femur BMSC | Increased mineral apposition rates, trabecular bone volume, number, thickness; decreased trabecular separation | Increased expression of ALP, OCN, RUNX2, OPG /// SMAD2 | |
| [19] | Inoue et al. | 2021 | Animal experiment | Physio ShockMaster, SAKAI Medical Co., Ltd., Tokyo, Japan | Rat osteoporotic model | Increased trabecular bone microarchitecture and bone strength | Decreased RANKL | |
| [20] | Inoue et al. | 2021 | Animal experiment | Physio ShockMaster, SAKAI Medical Co., Ltd., Tokyo, Japan | Rat osteoporotic fracture models | Increased bone volume/tissue volume | | Increased osteoblast surface, decreased number of sclerostin positive osteocytes |
| [21] | Zhao et al. | 2021 | Primary cell culture + animal experiment | Swiss DolorClast Master (Electro Medical Systems SA, Switzerland). | Human SBC-SPCs // rabbit osteochondral injury model | | Unaltered expression of OCN, RUNX2, COL2, SOX9; decreased expression of CEBPα and PPARγ; increased expression of YAP | Increased proliferation |
| [22] | Kobayashi et al. | 2020 | Animal experiment + secondary cell culture | Duolith® SD1-Storz Medical AG, Tagerwilen, Switzerland | Rat femur delayed union // ATDC5 cells | Increased bone union rate, radiographic score | | Increased enchondral ossification, chondrogenic differentation without inhibing proliferation |
| [23] | Alshihri et al. | 2020 | Primary cell culture | PiezoClast EMS, RICHARD WOLF GmbH, Knittingen, Germany | Goat iliac crest BMSC | | | Unaltered cell migration; increased proliferation and osteogenic differentation |
| [24] | Hsu et al. | 2020 | Animal experiment | Duolith® SD1-Storz Medical AG, Tagerwilen, Switzerland | Rat osteoporotic model | Increased bone strenght, bone mineral density, trabecular thickness, bone volume/tissue volume, porosity | | Increased expression of BMP2, BMP4, and Wnt3a signaling; unaltered expression of IGF1 |
| [25] | Ramesh et al. | 2020 | Animal experiment | Radial Spec., Medi Spec., Gaithersburg, MD, USA | Cultured fetal rat metatarsal bones | Increased bone length | | Increased number of proliferative chondrocytes of growth plate's cartilage and diameter of hypertrophic chondrocytes; activation of IGF1 and NFkb; increased levels of Bcl2 and Bcl-xL |
| [26] | Colbath et al. | 2020 | Primary cell culture | Pulse Veterinary Technologies, LLC | Equine BMSC | | Increased expression of ALP, decreased expression of TGFb, VEGF | |
| [27] | Hashimoto et al. | 2019 | Animal experiment | Dornier MedTech; Dornier ARIES Vet | Rat meniscus model | | Increased expression of COL2a1, ACAN, CCN2, SOX9 | Increased meniscal healing score and BrdU/CCN2-ratio |
| [28] | Senel et al. | 2019 | Animal experiment | Orthogold 100, MTS Medical, Konstanz, Germany | Rabbit osteotomy model | Bone mineral density, bone mineral content | | |
| [29] | Kim et al. | 2019 | Primary cell culture + animal experiment | Dornier AR2 electromagnetic head applicator | Rat chondrocytes // rat temporomandibular joint osteoarthritis model | Increased structure and bone quality | Decreased expression of TNFa, IL1b, IL6, MMP3, MMP13, BMP7 | Increased cell viability; decreased number of apoptotic cells and pro-inflammatory, cartilage degradation markers |
| [30] | Buarque de Gusmao et al. | 2019 | Animal experiment | Evotron, SwiTech Medical AG, Kreuzlingen, Switzerland (fok.) // MP 100, Storz Medical, Tägerwilen, Switzerland (rad.) | Rat tibial defects | | fESWT: increased Akt and FAK activity and TGFb1 expression<br>rESWT: increased FAK activity, decreased FAK activity | |
| [31] | Cheng et al. | 2019 | Animal experiment | Duolith® SD1-Storz Medical AG, Tagerwilen, Switzerland | Rat knee osteoarthritis model: subchondral bone / articular cartilage | Enhanced bone volume and trabecular thickness | | Reduced synovitis and cartilage damage, decreased expression of MMP-13, enhanced expression of RUNX-2, SOX-9 and Collagen Xα1, enhanced expression of IGF-1, TGF-β1, type II collagen and decreased TUNEL activity |
| [32] | Ginini et al. | 2019 | Animal experiment | Dermagold 100, Tissue Regeneration Technologies, Woodstock, GA, manufactured by MTS, Konstanz, Germany | Distraction osteogenesis in rats mandible: ESWT during distraction period | Increased mineral density, enhanced bone formation | | Higher collagen orientation index, expression of type I collagen and osteocalcin proteins |
| [33] | Ginini et al. | 2018 | Animal experiment | Dermagold 100, Tissue Regeneration Technologies, Woodstock, GA, manufactured by MTS, Konstanz, Germany | Distraction osteogenesis in rats mandible: ESWT during consolidation period | Higher degree of bone formation and mature bone, increased bone mineral density, bone volume fraction, and trabecular thickness | | Enhanced expression of bone morphogenetic protein-2, vascular endothelial growth factor, and proliferating cell nuclear antigen |
| [34] | Qi et al. | 2018 | Animal experiment | STORZ device (STORZ Medical, Tägerwilen, Switzerland) (radial) | Treatment of osteochondral defects of rabbit knees with scaffolds | Improved International Cartilage Repair Society (ICRS) score and macroscopic osteochondral appearance | | |
| [35] | Koolen et al. | 2018 | Animal experiment | Orthogold 180 c; MTS Medical, Konstanz, Germany | Screw fixation of rat cortical and cancellous bones | Cortical screws: increased bone formation and screw fixation; cancellous screws: no alterations | | |
| [36] | Mackert et al. | 2017 | Animal experiment | Duolith® SD1-Storz Medical AG, Tagerwilen, Switzerland | Osteoporotic fracture of rats tibia | Improved average stiffness and yield load | Increased expression of Collagen 1-alpha-1, Estrogen Receptor-α, Insulin-like Growth Factor 1, Osteocalcin, Tartrate-resistant Acid Phosphatase | Improved average ventral, dorsal and endosteal callus formation |
| [37] | Tan et al. | 2017 | Primary cell culture | KDE-2001 Extracorporeal Shockwave Lithotripter | HMSCs under 3D culture conditions | | ESWT alone: increased levels of A2B receptors; ESWT in combination with adenosine and A2BR-agonists downregulated ACAN, COL1A2, COL2A1, SOX9 and SOX6 | ESWT + adenosine and A2BR-agonists: inhibited chondrogenic differentation |
| [38] | Hsu et al. | 2017 | Animal experiment | not specified | Rat knee osteoarthritis model | | Increased expression of ERK1, OPG, ALP, MMP13; potential activation of the 1α,25-Dihydroxyvitamin D3 Rapid Membrane Signaling Pathway | Increased expression of Pdia-3 |
| [39] | Yilmaz et al. | 2017 | Animal experiment | Duolith® SD1-Storz Medical AG, Tagerwilen, Switzerland | Rat knee osteoarthritis model | Increased osteoblastic activity, improved pain score | | Lower modified Mankin score |
| [40] | Wang et al. | 2017 | Animal experiment | OssaTron (SANUWAVE Health, Inc., Alpharetta, GA) | Rat knee osteoarthritis model | Improved OARSI score and gross pathological changes, less cartilage defect, higher bone mineral density and bone volume, improved bone porosity and yield stress | | Increased expression PCNA and osteocalcin, decreased expression of TUNEL |
| [41] | Chen et al. | 2017 | Primary cell culture | Huikang type IV (Huikang, Shengzhen, China) | BMSC in osteogenic medium and implantation in mid-femur bone defects in rats | In vivo: improved bone volume, trabecular volume, BV/TV, bone thickness and bone mineral density | In vitro: increased expression of Col1, Runx2, osterix, and ALP | In vitro: enhanced proliferation and osteogenic differentiation; in vivo: increased bone formation and expression of Runx2 and Osterix |
| [42] | Onger et al. | 2017 | Animal experiment | OE050 focused applicator, Orthogold 100, MTS, Konstanz, Germany | Distraction osteogenesis in rats mandible: ESWT during consolidation period | 500 impulses per treatment: unaltered bone volume/bone density 1000 impulses per treatment: enhanced bone volume, bone density | | 500 impulses per treatment: enhanced capillary volume, decreased connective tissue volume<br>1000 impulses per treatment: enhanced capillary volume more positive areas of staining with VEGF, collagen antibody, BMP7 compared to control, but decreased capillary volume compared to 500 impulses; unaltered connective tissue volume; |
| [43] | Wang et al. | 2017 | Animal experiment | OssaTron (SANUWAVE Health, Inc., Alpharetta, GA) | Rat knee osteoarthritis model | Improved OARSI score and gross pathological changes, less cartilage defect, improved BV/TV ratio, improved bone porosity and trabecular thickness | | Decreased expression of TUNEL, higher amount PCNA-positive cells and increased vascular density; increased cartilage thickness and sectional cartilage area, decreased modified Mankin score |
| [44] | Lama et al. | 2017 | Animal experiment | Duolith® SD1-Storz Medical AG, Tagerwilen, Switzerland | Rat osteoporotic model | Prevention of bone weight reduction and trabecular microarchitecture deterioration; restored serum parameters of alkaline phosphatase, receptor activator of nuclear factor kappa-B ligand, osteoprotegerin, and PTH due to illness | Reduced cathepsin k, TNF-α levels, PPARγ and adiponectin transcription; increased runt-related transcripiton factor 2 and bone morphogenetic-2 expression | |
| [45] | Catalano et al. | 2017 | Primary cell culture | Piezoson 100, Richard Wolf, Knittlingen, Germany | Human adipose-derived mesenchymal stem cells | | Increased ERK phosphorylation, ROS formation, RUNX2, ALP, BMP2 | Increased Smad phosphorylation |

# Table S1: effects of the exposure of bone and cartilage tissue to extracorporeal shock waves

| Reference No. | Author | Year | Study type | Utilized device | Cell type investigated | Morphological, functional, radiological findings | Findings of molecularbiological examinations | Findings of histological examinations |
|---|---|---|---|---|---|---|---|---|
| [46] | Ma et al. | 2017 | Animal experiment | Orthospec (Medispec, Gaithersburg, MD, USA) | Rabbit necrotic femoral head model | Higher bone volume per tissue volume, trabecular thickness, trabecular number, osteoblast surface/bone surface, osteoid surface/bone surface, osteoid thickness, mineralizing surface/bone surface, mineralizing apposition rate, and bone formation rate as well as a reduced trabecular separation | | |
| [47] | Huang et al. | 2016 | Animal experiment | Orthospec™ (Medispec, Israel) | Osteoporotic fracture of rats tibia | | Increased expression of osteoprotegerin and bone morphogenetic protein-2 | |
| [48] | Notarnicola et al. | 2016 | Primary cell culture | Minilith SL1 (STORZ, Germany) | Human bone marrow stem cells | | Increased expression of BMP, alkalaine phosphatase, osteocalcin, COL1A1 and RUNX2 | Enhanced cell adhesion and proliferation |
| [49] | Zhai et al. | 2016 | Primary cell culture | MFL 5000 Lithotriptor, Dornier Medizintechnik, Wessling, Germany | Human bone mesenchymal stem cells from patients with avascular femoral head necrosis | | Increased expression of osteocalcin (OCN), core binding factor α1 (Cbfα1) and decreased peroxisome proliferator-activated receptor γ | Increased alkaline phosphatase content |
| [50] | Dias dos Santos et al. | 2015 | Animal experiment | EVOTRON-Vet® (SwiTech, Switzerland) | Bone drilled femurs of rats | | Increased contents of sulfated glycosaminoglycans and hyaluronic acid | |
| [51] | Wang et al. | 2014 | Animal experiment | OssaTron (SANUWAVE Health, Inc., Alpharetta, GA) | Rat osteoporosis and/or osteoarthritis model | Reduced arthritic area of injury joint, enhanced BMD and bone strength, improved subchondral plate thickness and bone porosity, reduced cartilage damage | | Increased Mankin and safranin O score, improved alterations of the molecular levels due to the illness of Dickkopf-1 (DKK-1), PCNA, VEGF, and BMP-2 |
| [52] | Muzio et al. | 2014 | Secondary cell culture | Piezoson 100, Richard Wolf, Knittlingen, Germany | Blocking of BMP of ESW-treated human osteoblast-like cells | | Decreased alkalaine phosphatase and osteocalcin | Increased cell growth |
| [53] | Oktas et al. | 2014 | Animal experiment | Stonelith PCK® lithotripter (Turkey, Ankara) | Fractures with both intact periosteum and excised periosteum in rats | No radiologic differences | | Excised periosteum group: positive effect on bone healing |
| [54] | Sun et al. | 2013 | Primary cell culture | KDE-2001 Extracorporeal Shockwave Lithotripter | Osteogenic differentation of human bone marrow mesenchymal stem cells | | Shockwave-dependent ATP release, that activated P2X7 receptors and downstream signaling events, which induced the differentation | |
| [55] | Suhr et al. | 2013 | Primary cell culture | Duolith®SD1 (Storz Medical AG, Trägerwilen, Switzerland) | Human bone marrow stromal cells | | | Extended growth rate, proliferation, migration, cell tracking and wound healing; ameliorated cell migration meditated by active remodeling of the actin cytoskeleton as indicated by increased directed stress fiber formations |
| [56] | Lyon et al. | 2013 | Animal experiment | not specified | Osteochondrosis dissecans of rabbit knees | Increased bony density | | More mature bone formation, better healing, higher density of the cartilage |
| [57] | Wang et al. | 2013 | Animal experiment | OssaTron (SANUWAVE Health, Inc., Alpharetta, GA) | Rat knee osteoarthritis model | Increased bone mineral density | | Improved Makin and Safranin O score, increased collagen II, decreased MMP13 |
| [58] | Wang et al. | 2013 | Animal experiment | OssaTron (SANUWAVE Health, Inc., Alpharetta, GA) | Rat knee osteoarthritis model | | | Treamtent 1-2 times per week: improved Makin and Safranin O score, increased collagen II, decreased MMP13, increased vWF, VEGF, BMP-2 and osteocalcin; deteriorated effects after 3 treatments per week |
| [59] | van der Jagt et al. | 2013 | Animal experiment | Dermagold, Tissue Regeneration Technologies, Woodstock, GA, manufactured by MTS, Konstanz, Germany | Rat osteoporotic model | Increased cortical volume (CtV), higher trabecular connectivity and, more plate like and thicker trabeculae, increased trabecular bone volume fraction | | |
| [60] | Oztemur et al. | 2013 | Animal experiment | Storz Masterpuls MP200 (STORZ, Germany) | Immature epiphysis of rats | No changes in bone length | | Increased blood vessel density, highly basophilic matrix and abundance of the differentiating chondrocytes |
| [61] | Gollwitzer et al. | 2013 | Animal experiment | Swiss Dolorclast® (Electro Medical Systems SA, Switzerland) | Rabbit femurs | New bone formation | | |
| [62] | Altuntas et al. | 2012 | Animal experiment | Storz Masterpuls MP200 (STORZ, Germany) | Rat subcondylar mandibular fracture model | | | Higher specimens' mean score in bone fracture healing |
| [63] | Notarnicola et al. | 2012 | Primary cell culture | enPuls, Zimmer MedizinSysteme GmbH, Germany | Murine calvaria osteoblasts | | Reduction in type 1 collagen, osterix, bone sialoprotein and receptor activator NF kappa ligand expression, osteocalcin, and osteopontin; in summary: inhibing effect on osteoclastogenesis | |
| [64] | Zhao et al. | 2012 | Animal experiment | Swiss Dolorclast® (Electro Medical Systems SA, Switzerland) | Rabbit knee osteoarthritis model | Decreased NO level, and severity of cartilage lesions | | Decreased chondrocyte apoptosis, enhanced Mankin score |
| [65] | Kearney et al. | 2012 | Animal experiment | OssaTron (SANUWAVE Health, Inc., Alpharetta, GA) | Rabbit tibia | | | Increased cambium cell number, cambium cell thickness, osteous tissue and callus area, larger amount of osteoprogenitor tissue; improved results in combination with a bioactive scaffold |
| [66] | Xu et al. | 2012 | Primary cell culture | Huikang type IV (Huikang, Shengzhen, China) | Rat osteoblasts | | Promotion of Integrin alpha-5 and beta-1-expression; induction of phosphorylation of the focal adhesion kinase, which led to an increased adhesion and migration of osteoblasts | |
| [67] | Wang et al. | 2012 | Animal experiment | OssaTron (SANUWAVE Health, Inc., Alpharetta, GA) | Osteoarthritis of rat knees | | | Improved Makin and Safranin O score, increased collagen II, VEGF, BMP-2 and osteocalcin expression |
| [68] | Ertürk et al. | 2012 | Animal experiment | Multimed Ortho (ElmedElectronics & Medical Industry & Trade Inc., Ankara, Turkey) | Intervertebral cartilage endplate in rabbits | No alterations in MRI | | Edema, increased fibroblastic activity, neovascularisation |
| [69] | Wang et al. | 2011 | Animal experiment | OssaTron (SANUWAVE Health, Inc., Alpharetta, GA) | Osteoarthritis of rat knees | Increased BMD, bone strength, modulus of elasticity | | Decreased Mankin score, improved Safranin O staining results, increased expression of VWF, VEGF, BMP2, OCN, ALP, decreased expression of CTXII, COMP |
| [70] | van der Jagt et al. | 2011 | Animal experiment | Dermagold; TissueRegeneration Technologies, Woodstock, Georgia, manufactured by MTS, Konstanz, Germany | Rat tibia | Increased 99mTc-MDP uptake, increased trabecular and cortical bone volume, higher bone stifness; no alterations in microcrack analysis | | Soft tissue damage, no periostal damage, de novo bone with active osteoblasts and osteoids |
| [71] | Notarnicola et al. | 2011 | Primary cell culture | Minilith SL1 (STORZ, Germany) | Murine calvaria osteoblast culture | | Increased expression of RUNX2, COL1, OCN, IGF-1, IGFBP-3; decreased expression of IGFBP-4 and -5 | |
| [72] | Hausdorf et al. | 2011 | Secondary cell culture | XL 1 (Dornier MedTech, Wessling, Germany) | Human osteoblasts and fibroblasts | | Increased FGF-2; no significant alterations in TGF-b | |
| [73] | Wang et al. | 2011 | Animal experiment | OssaTron (SANUWAVE Health, Inc., Alpharetta, GA) | Rabbit tibia acute fracture healing model | Increased BMC | | Increased bone tissue, decreased fibrous tissue; increased expression of VEGF, VWF, PCNA, OCN, BMP2, decreased expression of TUNEL |

# Table S1: effects of the exposure of bone and cartilage tissue to extracorporeal shock waves

| Reference No. | Author | Year | Study type | Utilized device | Cell type investigated | Morphological, functional, radiological findings | Findings of molecularbiological examinations | Findings of histological examinations |
|---|---|---|---|---|---|---|---|---|
| [74] | Mayer-Wagner et al. | 2010 | Animal experiment | XL 1 (Dornier MedTech, Wessling, Germany) | Rat knee | | Increased COL2A1 expression | Ultrastructural expansion of the rough-surfaced endoplasmatic reticulum, detachment of the cell membrane and necrotic chondrocytes; increased tenascin-C and Chi3L1; no alterations in Mankin Score |
| [75] | Muzio et al. | 2010 | Secondary cell culture | Piezoson 100, Richard Wolf, Knittlingen, Germany | Human osteoblast-like cells | | Increased expression of ALP, COL1, BMP-4, OCN | Increased osteoblast activity as well as number and size of calcium deposits |
| [76] | Lai et al. | 2010 | Animal experiment | OssaTron (High Medical Technology, Kreuzlingen, Switzerland) | Distraction osteogenesis of rat mandible | Treatment with 14kV: increased mineral densitiy, biomechanical bone strength, intense osteoblastic cell recruitment, new bone formation | | Treatment with 14kV: intense osteoblastic cell recruitment, new bone formation, neovascularisation, increased PCNA, VEGF, BMP-2; opposite effects after treatment with 21kV |
| [77] | Qin et al. | 2010 | Animal experiment | Dornier MedTech Epos, Wessling, Germany | Rabbit osteotendinous junction | Higher fraction of new bone | | Increased VEGF expression in hypertrophic chondrocytes, promotion of regeneration of the fibrocartilage zone |
| [78] | van der Jagt et al. | 2009 | Animal experiment | Dermagold/Orthowave 180, Tissue Regeneration Technologies, Woodstock, GA | Rat tibiae osteoporotic model | Diminished bone loss, higher trabecular bone volume fraction | | No differences in mineralization or osteoid appearance |
| [79] | Iannone et al. | 2009 | Primary cell culture | MINILITH SL1; Storz Medical, Kreuzlinger, Switzerland | Human osteoarthritic and healthy osteoblasts | | Increased expression of IL10, no alterations in TGFa, CD29, CD105 expression | |
| [80] | Tamma et al. | 2009 | Primary cell culture | MINILITH SL1; Storz Medical, Kreuzlinger, Switzerland | Murine calvaria osteoblasts | | Increased expression of Bax, RUNX2, OPN, BSP, OCN, COL1, decreased RANKL/OPG-ratio suggesting inhibition of osteoclasogenesis | |
| [81] | Lee et al. | 2009 | Animal experiment | OssaTron (High Medical Technology, Kreuzlingen, Switzerland) | Rabbit spinal fusion model | Increased callus formation and both extension and flexion stiffness | | |
| [82] | Tam et al. | 2009 | Animal experiment | Epos Ultra with EMSE O-140-AL source, Dornier MedTech, Wessling, Germany | Calcaneus of ovariectomized goats | Enhanced trabecular bone mineral density, trabecular bone volume fraction (BV/TV), trabecular thickness | | Increased mineral apposition rate |
| [83] | Hofmann et al. | 2008 | Primary cell culture | Sonocur Plus device (Siemens AG) | Primary human cancellous bone osteoblasts | | Altered expression of several genes involved in bone formation, osteoblast differentation and skeletal development; no alterations in Runx2, osterix, osteopontin, osteonectin, OC, TGFb1 expression | Enhanced mineralization and number of AP-positive osteoblasts |
| [84] | Tam et al. | 2008 | Primary cell culture | Dornier MedTech, Wessling, Germany | Human periosteal osteoblasts | | Decreased cell viability 6 days after treatment, increased viability 18 days after treatment; increased cell proliferation 18 days after treatment | Enhanced mineralization 35 days after treatment and AP activity 18 days after treatment |
| [85] | Lee et al. | 2008 | Animal experiment | OssaTron (High Medical Technology, Kreuzlingen, Switzerland) | Rabbits with dissociated transverse processes | New bone formation | | Superior fusion mass |
| [86] | Wang et al. | 2008 | Animal experiment | OssaTron (Sanuwave, Alpharetta, GA, USA) | Rabbit femur closed fracture model | Increased bone strength | | Increased cortical bone formation, higher number of neovessels, increased expression of VEGF, eNOS, PCNA, and BMP-2 |
| [87] | Moretti et al. | 2008 | Primary cell culture | MINILITH SL1; Storz Medical, Kreuzlinger, Switzerland | Human osteocytes: healthy and osteoarthritic | | Decreased expression x of IL-10, TNFa in both groups; no alteration in b1-integrin expression | |
| [88] | Tischer et al. | 2008 | Animal experiment | Epos + XL 1 (Dornier MedTech, Wessling, Germany) | Rabbit intact femur model | Dose-depending new bone formation | | Dose-depending new bone formation |
| [89] | Ozturk et al. | 2008 | Animal experiment | PCK, Stonalith Smart Lithotripter | Rabbit tibia epiphysis | | | Increased epiphyseal plaque thickness and number of chondrocytes |
| [90] | Ma et al. | 2007 | Animal experiment | Orthospec (Medispec, MD, USA) | Necrotic femoral head in rabbits | | Increased VEGF expression | Increased bone and osteoblast number; increased VEGF expression and microvessel density |
| [91] | Murata et al. | 2007 | Primary cell culture | Swiss Dolorclast® (Electro Medical Systems SA, Switzerland) | Rabbit chondrocytes | | Augmented uniform gene transfection and increased activity of vector-expressed genes | |
| [92] | Benson et al. | 2007 | Primary cell culture | pneumatically driven radial shock wave generator (Electro Medical Systems, Dallas, TX, USA) | Equine chondrocytes | | Decreased synthesis of GAG, no alterations in NO or PGE2 synthesis | |
| [93] | Martini et al. | 2006 | Secondary cell culture | Modulith SLX (Storz Medical AG, Kreuzlingen, Switzerland) + Ossatron (High Medical Technologies AG, Lengwil, Switzerland) | Human osteoblast-like cells (MG63) | | Dose- and device-dependent cell viability and expression of ALP, CICP, OCN, TGFb | |
| [94] | Bulut et al. | 2006 | Animal experiment | PCK, Stonalith Smart Lithotripter | Rabbits bony nonunion model | Increased callus volume | | Advanced bone healing |
| [95] | Martini et al. | 2005 | Secondary cell culture | Modulith SLX (Storz Medical AG, Kreuzlingen, Switzerland) | Human osteoblast-like cells (MG63) | Enhanced transmembrane current and voltage dependence of Ca-activated-/K- channels | | |
| [96] | Saisu et al. | 2005 | Animal experiment | Piezolith 2300 (Richard Wolf Inc., Knittlingen, Germany) | Acetabulum of immature rabbits | Increased breadth of the acetabular roof and transient woven bone formation on the lateral margin | | |
| [97] | Chen et al. | 2004 | Animal experiment | Ossatron (HMT High Medical Technologies GmbH, Kreuzlingen. Switzerland) | Rat segmental defect model | | Increased TGFb1 and VEGF-A expression | Increased cell density and cell number of RP59-pos. mesenchymal stem cells, subsequently enhanced differentation into chondrocytes and osteocytes |
| [98] | Saisu et al. | 2004 | Animal experiment | Piezolith 2300 (Richard Wolf Inc., Knittlingen, Germany) | Immature rabbit femurs | Enhanced bone mineral content, long-bone length and width | | |
| [99] | Chen et al. | 2004 | Animal experiment | Ossatron (HMT High Medical Technologies GmbH, Kreuzlingen. Switzerland) | Rat segmental defect model | | Increased ALPase, COL1, COL2, OCN expression and [3H]-thymidine uptake, increased expression and phosphorylation of ERK and p38 | Activated ERK and p38 expression |
| [100] | Pauwels et al. | 2004 | Ex vivo | Equitron (HMT High Medical Technologies, Kreuzlingen, Switzerland) // Swiss Dolorclast Vet (Electro Medical Systems, Dallas, TX, USA) | Equine cortical bone | No alterations in bone elasticity | | No histological alterations |
| [101] | Wang et al. | 2004 | Primary cell culture | not specified | Mesenchymal progenitor cells of human umbilical cord blood | | Induced superoxide production, enhanced TGFb1, RUNX2, OCN and COL1 expression, increased bone alkaline phosphatase activity | Increase in bone nodule formations, promotion of the CFU-Stroma formation but not CFU-Mix formation |
| [102] | da Costa Gomez et al. | 2004 | Ex vivo | Swiss DolorClast Vet Radial ESWT machine (Electro Medical Systems, Dallas, TX) + Equitron focused ESWT machine (High Medical Technologies Health Tronics Inc, Kennesaw, GA) | Ex-vivo metatarsal bones of horses | | | rESWT: increased microcrack length; fESWT: increased microcrack density |
| [103] | Takahashi et al. | 2004 | Animal experiment | Lithotripter A (Dornier MedTech, Munich, Germany) | Rat femur shafts | Increased cortical thickening, bone mineral density, bone mineral content | | Enhanced expression of COL1A1, COL2A1, OC, OPN, no alterations in expression of COL10A1 |
| [104] | Chen et al. | 2003 | Animal experiment | Ossatron (HMT High Medical Technologies GmbH, Kreuzlingen. Switzerland) | Rats segmental femoral defect model | Increased callus size and calcium content, bone mineral density | Increased AP activity, OCN production, PCNA, TGFb1 and BMP-2 expression | Increased bone tissue formation, progressive mesenchymal aggregation, enchondral ossification and hard callus formation |

# Table S1: effects of the exposure of bone and cartilage tissue to extracorporeal shock waves

| Reference No. | Author | Year | Study type | Utilized device | Cell type investigated | Morphological, functional, radiological findings | Findings of molecularbiological examinations | Findings of histological examinations |
|---|---|---|---|---|---|---|---|---|
| [105] | Martini et al. | 2003 | Primary cell culture | Ossatron (HMT High Medical Technologies GmbH, Kreuzlingen. Switzerland) | Primary sheep osteoblasts cultures | | High intensity treatment (28kV): decreased viability, detrimented cell respiration, depressed ALP and NO synthesis, decresed expression of OCN, TGFb and PICP; low intensity treatment (14kV) showed contrary effects with increased viability and cell respiration, increased ALP and NO synthesis as well as OCN and PICP expression; generally negative affection of PICP production | |
| [106] | Martini et al. | 2003 | Secondary cell culture | Ossatron (HMT High Medical Technologies GmbH, Kreuzlingen. Switzerland) | Human osteoblastlike cells | | Increased NO, OCN, TGFb1 production after low energy application (14kV); decreased cell viability and expression of all examined proteins at high application intensities (28kV) | |
| [107] | Dorotka et al. | 2003 | Primary cell culture | Modulith SLK (Storz Medical AG, Kreuzlingen, Switzerland) | Human articular chondrocytes and ovine bone marrow stromal cells | | Increased cytotoxity in both chondrocytes and BMSCs at high application intensities (0.17mJ/mm2) compared to lower energy levels and control; unaltered cell proliferation at all energy levels | |
| [108] | Wang et al. | 2003 | Animal experiment | Ossatron (HMT High Medical Technologies GmbH, Kreuzlingen. Switzerland) | Rat segmental femoral defect model | | Increased expression of BMP-2, BMP-3, BMP-4, and BMP-7 | Intensive mesenchymal cell aggregation, hypertrophic chondrogenesis, and endochondral/intramembrane ossification; increased levels of PCNA, BMP-2, BMP-3, BMP-4 |
| [109] | Maier et al. | 2002 | Animal experiment | Epos Ultra (Dornier MedTech, Wessling, Germany) | Intact distal rabbit femur | Scintigraphic decreased bone metabolism after 10 days, but increased metabolism after 28 days; signs of soft-tissue oedema, epiperiosteal fluid and bone-marrow oedema on MRI | | Epiperiostal deposits of hemosiderin |
| [110] | Wang et al. | 2002 | Animal experiment | Ossatron (HMT High Medical Technologies GmbH, Kreuzlingen. Switzerland) | Rat femur bone-marrow stromal cells | | Increased AP activity and TGFb1 expression | Promotion of bone marrow stromal, but not hematopoetic cell growth; dose-dependent effect on formation of CFU-osteoprogenitors |
| [111] | Wang et al. | 2001 | Secondary cell culture | Ossatron (HMT High Medical Technologies GmbH, Kreuzlingen. Switzerland) | Human bone marrow stromal cells | | Induction of cell membran hyperpolarization and consecutive Ras-activation, induction of osteogenic transcription factor CBFA1, increased activity of bone alkalaine poshphatase, increased expression of OCN, COL1 | Increased bone nodule formations |
| [112] | Wang et al. | 2001 | Animal experiment | Ossatron (HMT High Medical Technologies GmbH, Kreuzlingen. Switzerland) | Dog tibia facture model | More callus formations | | More cortical bone and thicker, denser, and heavier bone tissues |
| [113] | Väterlein et al. | 2000 | Animal experiment | Osteostar (Siemens,Erlangen, Germany) | Rabbit femur joint cartilage | Neither macroscopic nor radiological alterations after high intensity treatments | | No histological alterations after high intensity treatments |
| [114] | Peters et al. | 1998 | Primary cell culture | Siemens Lithostar Plus (Siemens, Erlangen, Germany) | Embryos of Oryzias latipes | | | Several damages of tissues after low intensity treatment |
| [115] | Augat et al. | 1995 | Animal experiment | XL2, Dornier Medical Systems, Munich, Germany | Ovine tibia fracture model | Neither alterations in biomechanical outcomes nor altered radiological results; tendency to deterioration of facture healing with increasing application intensities | | |
| [116] | Forriol et al. | 1994 | Animal experiment | Osteostar (Siemens,Erlangen, Germany) | Osteotomized lambs tibia | No effect on the periosteal surface of mature cortical bone, but on the endosteal surface induction of some new trabecular bone, delayed bone healing | | |
| [6] | Graff et al. | 1988 | Animal experiment | Dornier HM3 (Dornier Medical Systems, Munich, Germany) | Rabbit femur | Soft tissue bleeding | | Bone marrow hemorrhage and osteocyte damage 48h after ESWT; increased callus and bone formation, focal regeneration, apposition of new bone, bone remodeling |

# Table S2: effects of the exposure of connective tissue to extracorporeal shock waves

| Reference No. | Author | Year | Study type | Utilized device | Cell type investigated | Morphological, functional, radiological findings | Findings of molecularbiological examinations | Findings of histological examinations |
|---|---|---|---|---|---|---|---|---|
| [117] | Haberal et al. | 2021 | Animal experiment | Storz Masterpuls MP100 (STORZ, Germany) | Rat post-laminectomy | | | Decreased epidural fibrosis; unaltered acute/chronic inflammation and vascular proliferation |
| [118] | Heimes et al. | 2020 | Fertilized Chicken Eggs | Swiss Dolorclast® (Electro Medical Systems SA, Switzerland) | Fertilized chicken eggs.CAM-Assay | | Increased expression of MMP-9; decreased expression of MMP-13; unaltered expression of iNOS2, HIF-1α, VEGF | Increased coverage of the transplant by vasculature, percentage of the vascularized area, increase of the vascularized area and number of vessel junctions |
| [119] | Lu et al. | 2020 | Primary cell culture | Duolith®SD1 (Storz Medical AG, Trägerwilen, Switzerland) | Human ACL remnant cells + BMSC | | Increased ACL remnant cell viability; BMSC: increased expression of Ki67, COL1, COL3, unaltered expression of TGFb, VEGF | ACL-cells: increased expression of COL-I A1, TGF-β, and VEGF BMSC: increased migration and expression of EdU, COL1, COL3; unaltered expression of VEGF, TGFb |
| [120] | Basoli et al. | 2020 | Secondary cell culture | Orthogold 100, MTS Medical, Konstanz, Germany | Human foreskin fibroblasts | | Increased proliferation, ATP release, ROS production, expression of IL8, MCP1, HSP90, HSP27; unaltered expression of IL-6 | |
| [121] | Schnurrer-Luke-Vrbanić et al. | 2018 | Animal experiment | Swiss Dolorclast® / Piezoclast® (Electro Medical Systems SA, Switzerland) | Soft tissue regeneration in a rat fibula fracture model | | | Higher multiplication of collagen fibers; faster organisation of muscle fibers and vascularization by treatment with radial shockwaves |
| [122] | Cui et al. | 2018 | Primary cell culture | Duolith®SD1 (Storz Medical AG, Trägerwilen, Switzerland) | Human hypertrophic scar tissue fibroblasts | | Decreased expression of TGFb, a-SMA, vimentin, COL1a1, N-Cad, twist; increased expression of ID1, ID2, E-cad, FN after 24h, but decreased expression of FN after 72h | Decreased cell migration |
| [123] | Cai et al. | 2016 | Primary cell culture | Dermagold 100, Tissue Regeneration Technologies, Woodstock, GA, manufactured by MTS, Konstanz, Germany | Human periodontal ligament fibroblasts | | Initial decreased of IL-6, IL-8, MCP-1, TNF-α; after 4 and more hours: increase of IL6 and IL8, unaltered expression of MCP-1, TNF-α | |
| [124] | Hochstrasser et al. | 2016 | Secondary cell culture | Swiss Dolorclast® (Electro Medical Systems SA, Switzerland) | Human foreskin fibroblasts // human placental choriocarcinoma cells (JEG-3) | | Induced mechanical cell distruction, dose-dependent decreased cell viability, increased growth potential of fibroblasts (not of JEG-3 cells), shift in proportion from G0/G1 to G2/M phase in fibroblasts (not in JEG-3 cells) | Cellular detachments, holes in monolayers, disruption of actin filaments |
| [125] | Leone et al. | 2016 | Primary cell culture | Modulith SLK (Storz Medical AG, Kreuzlingen, Switzerland) | Human Tendon-derived Stem/Progenitor cells (hTSPCs) | | Increased expression of COL2A, SOX9, ALP, PPARy; unaltered expression of BGLAP, RUNX2 | Increased expression of differentiation markers in cells grown in specific differentiation media |
| [126] | Kisch et al. | 2015 | Animal experiment | Duolith®SD1 (Storz Medical AG, Trägerwilen, Switzerland) | Skin of the contralateral leg to the treated side in rats | Increased capillary blood velocity; unaltered postcapillary venous filling pressure | | |
| [127] | Waugh et al. | 2015 | Human experiment | Swiss Dolorclast® (Electro Medical Systems SA, Switzerland) | Microdialysate from peri-tendinous tissue | | Increased expression of IL-6, IL-8, MMP2 complex and ProMMP9; unaltered expression of (IL)-1β, IL-2, IL-4, IL-10, IL-12p70, IL-17A, VEGF, interferon-γ, Active MMP9, ProMMP2 and Active MMP2 | |
| [128] | de Girolamo et al. | 2014 | Primary cell culture | Orthogold 100, MTS Medical, Konstanz, Germany | Human tendon cells | | Increased expression of SCX, IL-1β, IL-6, IL-10 , TGFβ , VEGF; unaltered expression of MMP-3, MMP-13, COL1 A1, COL3 A1, and TNFα; reduced NO synthesis | |
| [129] | Chow et al. | 2014 | Animal experiment | Epos (Dornier MedTech, Wessling, Germany) | Rabbit delayed tendon bone insertion healing | | | Increased fibrocartilage area and thickness, proteoglycan deposition, expression of SOX9, COLII, Vickers hardness; unaltered expression of COL1 |
| [130] | Cinar et al. | 2013 | Animal experiment | Swiss Dolorclast® (Electro Medical Systems SA, Switzerland) | Rat Achilles tendinitis | Decreased Load to failure | | Decreased collagen fiber density |
| [131] | Contaldo et al. | 2012 | Animal experiment | Swiss Dolorclast® (Electro Medical Systems SA, Switzerland) | Mice wound healing model | | | Enhanced expression of caspase-3, PCNA, eNOS; increase of functional angiogenetic density and total wound score |
| [132] | Chow et al. | 2012 | Animal experiment | Epos (Dornier MedTech, Wessling, Germany) | Rabbit delayed tendon bone insertion healing | Increased Load to failure, new bone area and new bone volume | | Increased fibrocartilage zone and ratio of bone forming |
| [133] | Yoo et al. | 2012 | Animal experiment | Dornier AR2 (Dornier MedTech, Wessling, Germany) | Rat collagenase-induced achilles tendinitis | | | Increased fibrillary diameter, vascularity, fibroblast activity, lymphocyte and plasma cell infiltration, dense histocytes; transient disorganization of collagen fibers |
| [134] | Leone et al. | 2012 | Primary cell culture | Modulith SLK (Storz Medical AG, Kreuzlingen, Switzerland) | Human ruptured / healthy tenocytes | | Ruptured tenocytes: decreased expression of COL1, SCX; unaltered COL3, TNM, TN-C | Healthy tenocytes: increased cell proliferation and migration |
| [135] | Zhang et al. | 2011 | Animal experiment | OssaTron + EvoTron (SANUWAVE, Alpharetta, GA, USA) | Rat various tendons and septa | | | Increased lubricine expression |
| [136] | Penteado et al. | 2011 | Animal experiment | OssaTron® (Barueri, SP, Brazil) | Rabbit Patellar tendon | | | Unaltered blood vessel number |
| [137] | Kubo et al. | 2010 | Animal experiment | Medispec Ltd, Germantown, Md | Rabbit ear lymphedema | Reduced ear thickness | Increased expression of VEGF-C, VEGF-R3 | Increased density of lymphatic vessels |
| [138] | Sugioka et al. | 2010 | Primary cell culture | Swiss Dolorclast® (Electro Medical Systems SA, Switzerland) | Rat Achilles tendon cells | | | Increased introduction of NF-κB decoy-FITC, activation of NF-κB; decreased activation of NF-κB after pretreatment with ESW+NF-κB decoy-FITC |
| [139] | Berta et al. | 2009 | Secondary cell culture | Piezoson 100, Richard Wolf, Knittlingen, Germany | Human fibroblasts | | Decreased viability; increased expression of TGF−β1; increase of COL1 and COL3 expression after 6 days after a primary decreased expression | |
| [140] | Bosch et al. | 2009 | Animal experiment | Equitron, High Medical Technologies, Lengwil, Switzerland | Various pony tendons | | Increased expression of COL1 and MMP14; decreased expression of MMP3 | Unaltered total collagen content, disorganisation of normal collagen structure; decreased percentage of degraded collagen 6 weeks after treatment after an increase 3h after treatment |
| [141] | Han et al. | 2009 | Primary cell culture | Sonocur (Siemens Medical Solutions, Iselin, NJ) | Healthy and diseased tenocytes | | Healthy: increased expression of IL1; unaltered expression of MMP1, MMP2, MMP9, MMP13, IL6 and IL13<br>diseased: decreased expression of MMP1, MMP13 and IL6; unaltered expression of MMP2, MMP9, IL1 and IL13 | Decreased cell viability |
| [142] | Byron et al. | 2009 | Animal experiment | Swiss DolorClast Vet, EMS Electro Medical Systems, Nyon, Switzerland | Palmar heel pain in horses | Radiographic scores, scintigraphic navicular pool phase, delayed phase ROI density ratios | | |
| [143] | Chao et al. | 2008 | Primary cell culture | Dornier Electromagnetic Shock Wave Emitter (EMSE 220F; Dornier MedTech GmbH, Wessling, Germany) | Rat tenocytes | | Increased total collagene concentration, NO production, expression of PCNA, COL1, COL3, TGFb | Decreased cell viability; increased cell proliferation |
| [144] | Wang et al. | 2008 | Animal experiment | Epos (Dornier MedTech, Wessling, Germany) | Rabbit Patella-patellar tendon healing junction | Increased new bone formation, bone mineral status, tensile load and strength | | Increased remodeling / alignment of collagen fibers, thicker and mature regenerated fibrocartilage zone |
| [145] | Bosch et al. | 2007 | Animal experiment | Equitron, High Medical Technologies, Lengwil, Switzerland | Various pony tendons | | Unaltered DNA content, 3h after treatment: increased GAG, total protein synthesis; 6weeks after treatment: decreased GAG, collagen synthesis, noncollagenous protein synthesis, total protein synthesis | Unaltered total collagen content, disorganisation of normal collagen structure; decreased percentage of degraded collagen 6 weeks after treatment after an increase 3h after treatment |
| [146] | Kersh et al. | 2006 | Animal experiment | Equitron, High Medical Technologies, Lengwil, Switzerland | Horse superficial digital flexor tendinitis model | | | Unaltered percentage lesion, percentage disruption and grey scale, external width, fibroblast and tenocyte number, increased capillary density |

# Table S2: effects of the exposure of connective tissue to extracorporeal shock waves

| Reference No. | Author | Year | Study type | Utilized device | Cell type investigated | Morphological, functional, radiological findings | Findings of molecularbiological examinations | Findings of histological examinations |
|---|---|---|---|---|---|---|---|---|
| [147] | Wang et al. | 2005 | Animal experiment | Ossatron (HMT High Medical Technologies GmbH, Kreuzlingen. Switzerland) | Rabbit tendon-bone interface healing | Increased trabecular bone around the tendons and tensile strength of tendon/bone-interface, better bone/tendon contacting | | |
| [148] | Chen et al. | 2004 | Animal experiment | HMT High Medical Technologies GmbH, Kreuzlingen, Switzerland | Rat Achilles tendinitis | Increased load to failure | | Decreased edema, swelling, inflammatory cell infiltration; increased expression of TGF-b, IGF-I, tenocyte proliferation, neovascularization and progressive tendon tissue regeneration |
| [149] | Orhan et al. | 2004 | Animal experiment | Breakstone 200 Lithotripter System (Breakthrough Medical Corp, Gaittersburg, Maryland) | Rat Achilles tendon injury | Higher force to rupture | | Less adhesion formation, increased number of capillaries |
| [150] | Hsu et al. | 2004 | Animal experiment | Orthospec, Medispec, Israel | Rabbit patellar tendinopathy | Increased ultimate tensile load | | Increased hydroxyproline concentration; decreased pyridinoline concentration; unaltered number of blast-like tenocytes (4 weeks); increased number of mature tenocytes (16 weeks) |
| [151] | Orhan et al. | 2004 | Animal experiment | mobile ELMED lithotriptor, 2001, Ankara, Turkey | Rat healthy Achilles tendon | | | Disorganisation of collagen fibers |
| [152] | Wang et al. | 2003 | Animal experiment | not specified | Rabbit Achilles tendon-bone junction | | | Increased number of neo-vessels and expression of eNOS,VEGF and PCNA |
| [153] | Maier et al. | 2002 | Animal experiment | EPOS Ultra, Dornier MedTech, Wessling,Germany | Rabbit Quadriceps tendon | | | High intensities: tendon: increased staining affinity, nuclear and fibrillar appearance paratendon: increased thickness, edema, capillary density |
| [154] | Wang et al. | 2002 | Animal experiment | Ossatron (HMT High Medical Technologies GmbH, Kreuzlingen. Switzerland) | Dog Achilles tendon bone junction | | | New capillary and muscularized vessels, newly appeared myofibroblasts; no alterations in bone matrix, bone vascularization and osteocyte activity |
| [155] | Johannes et al. | 1994 | Cell culture | Osteostar (Siemens,Erlangen, Germany) | Human fibroblasts | | | Decreased cell viability, no alterations in cell growth |

# Table S3: effects of the exposure of muscle and nerve tissue to extracorporeal shock waves

| Reference No. | Author | Year | Study type | Utilized device | Cell type investigated | Morphological, functional, electrophysiological findings | Findings of molecularbiological examinations | Findings of histological examinations |
|---|---|---|---|---|---|---|---|---|
| [156] | Huang et al. | 2021 | Animal experiment | Swiss Dolorclast® (Electro Medical Systems SA, Switzerland) | Rabbit immobilization-induced fibrosis and contracture of muscle | Decreased total contracture angle, muscle contracture angle | Decreased expression of TGFb, HIF1a | Decreased proportion of collagen fiber area |
| [157] | Kenmoku et al. | 2021 | Animal experiment | Swiss Dolorclast® (Electro Medical Systems SA, Switzerland) | Neuromuscular junction in rats | Energy flux density- and total energy-dependent decrease of CMAP, unaltered CMAP latency | | |
| [158] | Park et al. | 2020 | Animal experiment | Dornier MedTech, Wessling, Germany | Rat sciatic nerve crush model | Increased print width, print area | Tendential increased expression of myelin basic protein | |
| [159] | Matsuda et al. | 2020 | Animal experiment | Duolith®SD1 (Storz Medical AG, Trägerwilen, Switzerland) | Spinal cord contusion injury model in rats | Improved BBB locomotor function, increased withdrawal threshold, abbreviated latency of MEPs, no alterations in MEP amplitude | Increased expression of BDNF and TrkB | Increased expression of BDNF, reduced myelin damage and oligodendrocyte loss, decreased axonal damage |
| [160] | Langendorf et al. | 2020 | Animal experiment | Swiss Dolorclast® (Electro Medical Systems SA, Switzerland) | Rat musculus rectus femoris grade III muscle tear | | Increased expression of MyoD and myosin | First higher amount of mononucleated cells, at day 7 newly formed muscle fibers with less MNCs; unaltered number of CD31-pos. cells. |
| [161] | Sagir et al. | 2019 | Animal experiment | OE050 focused applicator, Orthogold 100, MTS, Konstanz, Germany | Rat peripheral nerve injury | Decreased EMG amplitude, increased EMG latency, improved sciatic functional index | | Decreased Myelin thickness, axon area and number |
| [162] | Feichtinger et al. | 2019 | Animal experiment | Dermagold; TissueRegeneration Technologies, Woodstock, Georgia, manufactured by MTS, Konstanz, Germany | Rat rotator cuff reconstruction | Improved load-to-failure testing results, intensity measurements in functional gait analysis | Unaltered expression of CXCL12, TGF-β1, TGF-β3, VEGFR2 | |
| [163] | Yang et al. | 2019 | Animal experiment | not specified | Neuropathic pain // rat L4/5 dorsal root ganglions and sciatic nerve | Improved mechanical paw withdrawal treshold and thermal paw withdrawal latency | Decreased TNF-α, NF-κB, MMP-9, IL-1ß, NOX-1, NOX-2, NOX-4, oxidized protein, cleaved caspase 3, cleaved PARP, γ-H2AX, (p)-p38, p-JNK, p-ERK1/2, Nav.1.3, Nav.1.8 and Nav.1.9 | |
| [164] | Mattyaszovszky et al. | 2018 | Primary cell culture | Swiss Dolorclast® (Electro Medical Systems SA, Switzerland) | Human sceletal muscle cells | | Dose-dependent increase of Myf5, MyoD, Pax7, NCAM; down-regulation of these proteins at double exposure of the highest energy flux density | Increased cell viability at low energy flux densities, no alterations at higher energy flux densities |
| [165] | Yin et al. | 2018 | Animal experiment | Cardiospec, Medispec, Israel | Rat limb ischemia-reperfusion injury of quatriceps muscle | Increased angiogenesis, decreased serum myoglobin/creatine phosphokinase | Decreased NOX-1, NOX-2, cleaved caspase 3, cleaved PARP, TGF-β, (p-)Smad3, ICAM-1, MMP-9, TNF-α, NF-κB, RANTES, TLR-2, TLR-4, IL-1β, cytosolic cytochrome C, γ-H2AX; increased Bcl-2, p-Smad1/5, BMP-2, mitochondrial cytochrome C | Decreased muscle-damaged/fibrosis/collagen-deposition areas |
| [166] | Shin et al. | 2018 | Animal experiment | Swiss Dolorclast® (Electro Medical Systems SA, Switzerland) | Rat chronic contusive SCI model | | Increased expression of DCX, SOX-2, GAP-43, MAP-2 | Increased expression of DCX, SOX-2, GAP-43, MAP-2 |
| [167] | Luh et al. | 2018 | Animal experiment | Piezoson 100, Richard Wolf, Knittlingen, Germany | Rat caudal nerves anaesthetic drug delivery | | | |
| [168] | Kenmoku et al. | 2018 | Animal experiment | Swiss Dolorclast® (Electro Medical Systems SA, Switzerland) | Rat neuromuscular junction | Decreased CMAP amplitude, unaltered CMAP latency | | Irregular end plates, unchanged axon terminals and muscle fibers, increased mean interjunctional fold interval |
| [169] | Chen et al. | 2017 | Animal experiment | not specified | Neuropathic pain // rat L4/5 dorsal root ganglions and sciatic nerve | Improved mechanical paw withdrawal treshold and thermal paw withdrawal latency | Decreased expression of TNF-α, NF-κB, MMP-9, IL-1ß, GFAP, ox42, NOX-1, NOX-2, NOX-4, oxidized protein, γ-H2AX, cytosolic mitochondria, cleaved capase-3, PARP, p-P38, p-JNK, p-ERK1/2, Nav.1.3, Nav.1.8, Nav.1.9 | Decreased expression of p-P38+, peripherin+ cells, P38+, NF200+ cells |
| [170] | Yahata et al. | 2016 | Animal experiment | Duolith®SD1 (Storz Medical AG, Trägerwilen, Switzerland) | Rat SCI model | Improved BBB locomotor score, withdrawal latency, 50% withdrawal threshold | | Increased expression of VEGF, CD31, a-SMA, 5-HT, increased area of spared white matter, decreased number of TUNEL-pos. cells |
| [171] | Schuh et al. | 2016 | Primary cell culture | Dermagold 100, Tissue Regeneration Technologies, Woodstock, GA, manufactured by MTS, Konstanz, Germany | Schwann cells of rats peripheral nerves(sciatic nerves) | | Increased cell yield, BrdU assays, population doublings, S100b, c-Jun, GFAP, and P75 expression, decreased P0 and P16 expression, increased extracellular ATP levels immidiately after application | |
| [172] | Lee | 2016 | Animal experiment | HAEMIL, Soltar, Korea | Locomotion in a rats CNS injury model | Decreased knee joint angle | | |
| [173] | Kisch et al. | 2016 | Animal experiment | Duolith®SD1 (Storz Medical AG, Trägerwilen, Switzerland) | Rat hind limb muscle | Increased muscular blood flow | | |
| [174] | Lee & Kim | 2015 | Animal experiment | HAEMIL, Soltar, Seoul, Korea | Rat sciatic nerve injury | Increased ankle angles (toe off + foot contact), improved Sciatic functional index | Increased expression of NT-3 | |
| [175] | Yamaya et al. | 2014 | Animal experiment | Duolith®SD1 (Storz Medical AG, Trägerwilen, Switzerland) | Rat SCI model | Improved BBB locomotor score | Increased expression of VEGF and Flt-1 | Increased NeuN-positive cells, VEGF staining |
| [176] | Fu et al. | 2014 | Animal experiment | Storz Masterpuls MP100 (STORZ, Germany) | Neuropathic pain / rat chronic constriction injury | Improved mechanical withdrawal threshold, thermal withdrawal latency | | |
| [177] | Ishikawa et al. | 2013 | Animal experiment | Swiss Dolorclast® (Electro Medical Systems SA, Switzerland) | Rat CCI hind limb muscle | Transfection of POMC gene | | |
| [178] | Mense & Hoheisel | 2013 | Animal experiment | Duolith®SD1 (Storz Medical AG, Trägerwilen, Switzerland) | Rat distal sciatic nerve injury (Gastrocnemius–Soleus or Sural Nerve) | Decreased pressure pain threshold, improved locomotor activity | | Increased number of PGP 9.5 IR nerve fibers |
| [179] | Hausner et al. | 2012 | Animal experiment | Orthowave 180 shockwave machine (MTS Europe,Switzerland) | Rat sciatic nerve autograft | Increased amplitude, CNAP area | | Increased number of myelinated axons, unaltered number of endoneural vessels |
| [180] | Kenmoku et al. | 2012 | Animal experiment | Swiss Dolorclast® (Electro Medical Systems SA, Switzerland) | Rat gastrocnemius muscle neuromuscular junction | Decreased amplitude, unaltered CMAP latency | | Decreased number of acetylcholine receptors |
| [181] | Yamashita et al. | 2009 | Primary cell culture + animal experiment | not specified | POMC transfection in rat muscle cells | Decreased mechanical allodynia | | Increased ratio of β-endorphin-IR muscle cells and number of β-endorphin-IR muscle fibers; decreased number of CGRP-IR DRG neurons |
| [182] | Wu et al. | 2008 | Animal experiment | Piezoson 100, Richard Wolf, Knittlingen, Germany | Rat sciatic nerve | Decreased motor nerve conduction velocity; unaltered Sciatic functional index and withdrawal reflex latency | | Damage to the myelin sheath of large-diameter myelinated fibers |
| [183] | Hausdorf et al. | 2008 | Animal experiment | XL 1 (Dornier MedTech, Wessling, Germany) | Rabbit distal femoral region | | | Decreased number of unmyelinated nerve fibers of femoral nerve; unaltered number of unmyelinated nerve fibers of sciatic nerve; unaltered size, number and myelin sheet of myelinated nerve fibers |
| [184] | Hausdorf et al. | 2008 | Animal experiment | XL 1 (Dornier MedTech, Wessling, Germany) | Rabbit distal femoral region // dorsal root ganglion | | | Decreased number of neurons immunoreactive for substance P |
| [185] | Lee et al. | 2007 | Animal experiment | Ossatron (HMT High Medical Technologies GmbH, Kreuzlingen. Switzerland) | Rabbit spinal cord | No changes in motor and vegetative functions | | Decreased number of neurons at high intensity treatment (0.68mJ/mm2), dose-dependent myelin damage |
| [186] | Ochiai et al. | 2007 | Animal experiment | Epos (Dornier MedTech, Wessling, Germany) | Rat knee osteoarthritis // dorsal root ganglion | Increased walking duration | | Decreased ratio of CGRP-positive dorsal root ganglion neurons |
| [187] | Wu et al. | 2007 | Animal experiment | Piezoson 100, Richard Wolf, Knittlingen, Germany | Rat sciatic nerve | Decreased motor nerve conduction velocity, unaltered sciatic functional index | | |
| [188] | Murata et al. | 2006 | Animal experiment | Epos (Dornier MedTech, Wessling, Germany) | Rat foot pad // dorsal root ganglion | | | Increased number of ATF3 and ATF-3/GAP-43 dual-IR neurons |

# Table S3: effects of the exposure of muscle and nerve tissue to extracorporeal shock waves

| Reference No. | Author | Year | Study type | Utilized device | Cell type investigated | Morphological, functional, electrophysiological findings | Findings of molecularbiological examinations | Findings of histological examinations |
|---|---|---|---|---|---|---|---|---|
| [189] | Takahashi et al. | 2006 | Animal experiment | Epos (Dornier MedTech, Wessling, Germany) | Rat foot pad | | | Decreased number of epidermal nerve fibers |
| [190] | Bolt et al. | 2004 | Animal experiment | Swiss DolorClast Vet, EMS Electro Medical Systems, Nyon, Switzerland | Horse digital palmar nerves | Decreased sensory nerve conduction velocity | | Disruption of myelin sheet |
| [191] | Hausdorf et al. | 2004 | Animal experiment | XL 1 (Dornier MedTech, Wessling, Germany) | Rabbit periosteoum of distal femur | | Increased Substance P release 6 and 24 hours after treatment, decreased Substance P release 6 weeks after treatment; unaltered Prostaglandine E release | |
| [192] | Takahashi et al. | 2003 | Animal experiment | Epos (Dornier MedTech, Wessling, Germany) | Rat foot pad // dorsal root ganglion | | | Decreased percentage of CGRP-immunoreactive dorsal root ganglion neurons |
| [193] | Maier et al. | 2003 | Animal experiment | XL 1 (Dornier MedTech, Wessling, Germany) | Rabbit distal femur | | | Increased Substance P release after 6 and 24 hours; decreased SP release after 6 weeks; no alterations in PGE2 release |
| [194] | Haake et al. | 2002 | Animal experiment | MINILITH SL1; Storz Medical, Kreuzlinger, Switzerland | Rat hind paw // spinal chord | | Unaltered c Fos expression | Unaltered c Fos expression |
| [195] | Ohtori et al. | 2002 | Animal experiment | Epos (Dornier MedTech, Wessling, Germany) | Rat hind paw epidermis | | | Decreased number of nerve fibres immunoreactive for PGP 9.5 and CGRP |
| [196] | Haake et al. | 2001 | Animal experiment | MINILITH SL1; Storz Medical, Kreuzlinger, Switzerland | Rat hind paw // spinal chord | | | Unaltered expression of met-enkephalin (MRGL) and dynorphin (Dyn) |
| [197] | Rompe et al. | 1998 | Animal experiment | Osteostar (Siemens,Erlangen, Germany) | Rabbit sciatic nerve | | | Vacuolic swelling of the axons, no disruption of nerve's continuity |
